# Effect of Xinyue capsules on patients with coronary heart disease after percutaneous coronary intervention: study protocol for a randomized controlled trial

**DOI:** 10.1186/s13063-016-1531-x

**Published:** 2016-08-18

**Authors:** Ming Guo, Ming-jie Zi, Rui-xi Xi, Qiao-ning Yang, Rui-na Bai, Yi-sheng Zhang, Yu-hua Wang, Pei-li Wang, Da-zhuo Shi

**Affiliations:** 1 Cardiovascular Diseases Center, Xiyuan Hospital, China Academy of Chinese Medical Sciences, 1 Xiyuan Caochang, Haidian District, Beijing, 100091 China; 2 Department of Medicine, Jilin Jilin Yisheng Pharmaceutical Co., Ltd., 17 Wen Hua Dong Lu Road, Ji’an, 134200 China

**Keywords:** Xinyue capsule, Coronary heart disease, Randomized controlled trial

## Abstract

**Background:**

The risk of cardiovascular events remains high in patients with coronary heart disease (CHD) after successful percutaneous coronary intervention (PCI). *Panax quinquefolius* saponin, a major component of Xinyue capsule, has been used to treat patients with CHD. The aim of this study is to evaluate the efficacy and safety of Xinyue capsules in patients with CHD after PCI.

**Methods/design:**

This study is a multicenter, placebo-controlled, double-blind, randomized controlled clinical trial. A total of 1100 participants are randomly allocated to two groups: the intervention group and a placebo group. The intervention group receives Xinyue capsules plus conventional treatment, and the placebo group receives placebo capsules plus conventional treatment. The patients receive either Xinyue or placebo capsules three times daily (1.8 g/day) for up to 24 weeks. The primary outcome measure is the time from randomization to the first occurrence of major adverse cardiovascular events. The secondary outcome measure is the time from randomization to the first occurrence of stroke, pulmonary embolism, and peripheral vascular events, as well as death due to any cause. All outcome measures will be assessed at 12, 24, 36, and 48 weeks after randomization. Adverse events will be monitored during the trial.

**Discussion:**

The aim of this study is to evaluate the effects of Xinyue capsules on patients with CHD after interventional treatment. The results of this trial will provide critical evidence regarding Chinese herbal medicine treatment for CHD.

**Trial registration:**

Chinese Clinical Trials Registry identifier ChiCTR-IPR-14005475. Registered on 10 November 2014.

**Electronic supplementary material:**

The online version of this article (doi:10.1186/s13063-016-1531-x) contains supplementary material, which is available to authorized users.

## Background

Percutaneous coronary intervention (PCI) is commonly performed for coronary revascularization in patients with stable angina or acute coronary syndrome (ACS) [1]. Nevertheless, there are several medications with proven benefit to patients with cardiovascular disease, such as dual-antiplatelet therapy, statins, β-blockers, and angiotensin-converting enzyme inhibitors [2]. Unfortunately, the risk of cardiovascular events remains high in patients after PCI [3]. Many patients do not receive the conventional treatment due to the side effects, contraindications, and drug-drug interactions [4]. Selecting the optimal clinical strategies to prevent the occurrence of cardiovascular events is challenging.

Traditional Chinese medicine (TCM) has been used to treat coronary heart disease (CHD) for thousands of years [5]. From the perspective of TCM, patients with CHD can be divided into different syndromes (i.e., different *zhengs*). In the diagnosis of CHD, the “Qi and Yin inadequacy syndrome” is the important subtype. In 2005, Xinyue capsules were approved by the China Food and Drug Administration for treatment of CHD. The main component of Xinyue capsules—*Panax quinquefolius* saponin (PQS)—is extracted from the stem and leaves of *Panax quinquefolium*, which in TCM theory could reinforce Qi and nourish Yin. Previous studies have shown that PQS has various pharmacological actions, including anti-myocardial cell damage [6], protection of heart function, reduction of myocardial oxygen consumption [7, 8], improvement of myocardial perfusion [9] and ventricular remodeling after acute myocardial infarction (MI) [10], antiapoptosis of ischemic myocardial cells [11, 12], regulation of glucose and lipid metabolism [13], and improvement of insulin resistance [14, 15]. A multicenter randomized clinical study [16] showed that the combination of Xinyue capsules with Chuanxiong capsules and conventional Western interventions could reduce the occurrence of cardiovascular events in patients with ACS after PCI without increasing the risk of major bleeding. The results of pharmaceutical chemistry and pharmacokinetics conducted jointly by Xiyuan Hospital and Medical University of Vienna showed that the fingerprint of Xinyue capsules from different batches or the same batch at different time points was consistent, and the measurements of heavy metal and pesticide residues were within European Union standards.

Based on the multifactorial effect on CHD of Xiyue capsule, our hypothesis is that Xiyue capsules plus conventional treatment can improve cardiovascular outcomes in patients with CHD after interventional treatment compared with conventional treatment alone. If successful, it will provide a novel, promising alternative strategy for further reducing cardiovascular events.

## Methods/design

### Study design

This study is registered in the Chinese Clinical Trials Registry (ChiCTR-IPR-14005475). It is a multicenter, double-blind, randomized, placebo-controlled clinical trial. This study complies with the principles of the Declaration of Helsinki and Good Clinical Practice guidelines. Written informed consent will be obtained from all patients prior to their participation in this study, and the recruited patients will be randomized to either the Xiyue capsule group or the placebo group. We will rigorously follow the Consolidated Standards of Reporting Trials (CONSORT) recommendations in reporting the results [17].

**Fig. 1:**
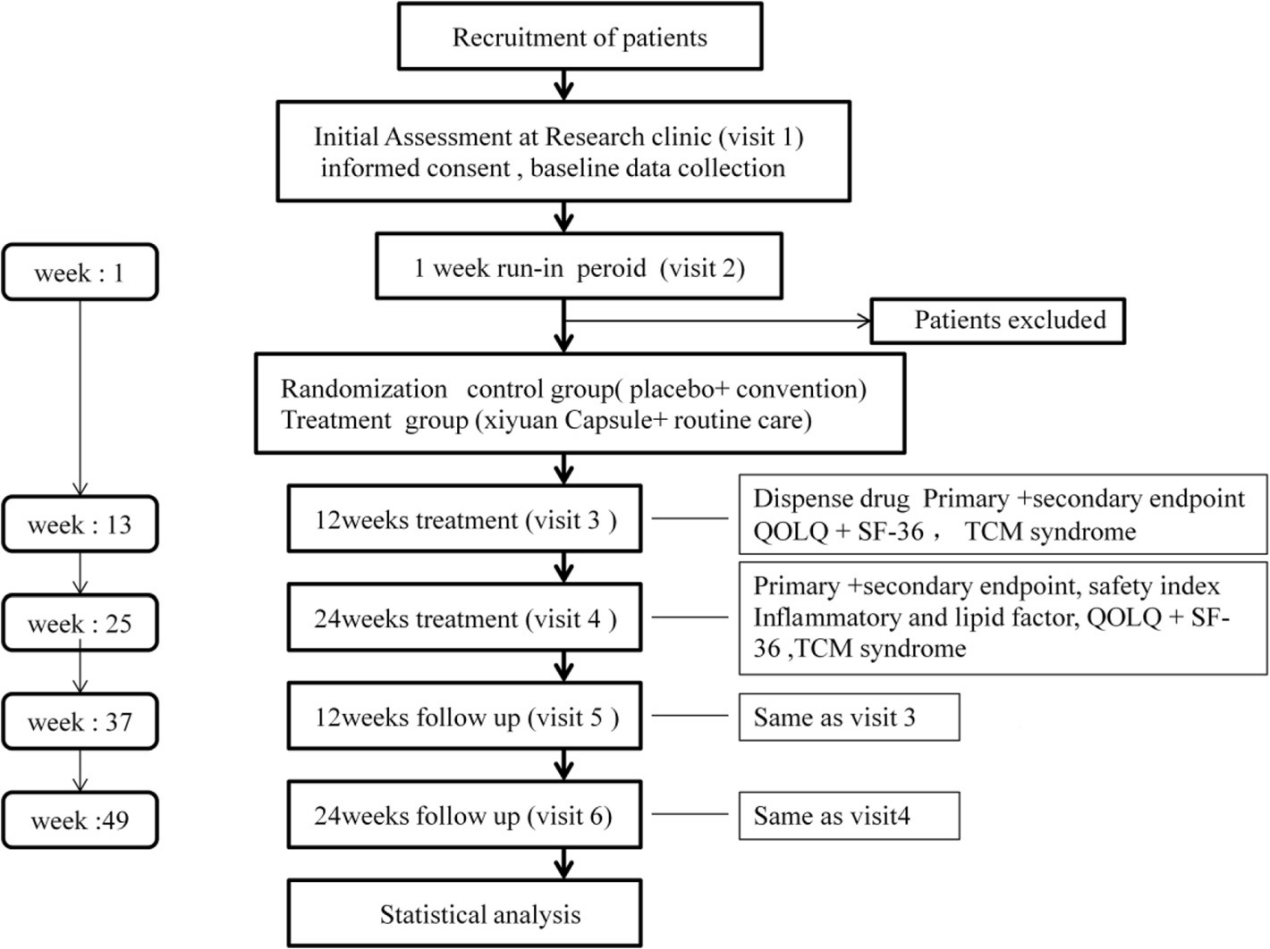
Study flowchart. *QOLQ* Quality of Life Questionnaire, *SF-36* 36-item Short Form Health Survey, *TCM* traditional Chinese medicine

The trial will be conducted in 25 centers in China (see in ​Additional file 1). A total of 1100 participants will be recruited. After acquiring consent from the participants or their parents and/or legal guardians, the participants will be enrolled in the trial, which consists of a 1-week run-in period, a 24-week treatment period, and a 24-week follow-up period. An outline of the study procedures is illustrated in Fig. 1.

### Participants

Inpatients and outpatients in the participating centers will be screened if they meet the selection criteria.

#### Inclusion criteria

1. CHD was diagnosed according to the guidelines [2, 18]. All participants must have received successful PCI.
2. The time window is within 1 year after PCI and the patient must be clinically stable, defined as follows:
  - No progressive chest pain is present.
  - The troponin value is within normal range or slightly elevated, but lower than the 99th percentile of the upper reference limit value.
  - The participants must have a Canadian Cardiovascular Society classification of angina I to II
3. Patients are diagnosed as having a syndrome of deficiency of both Qi and Yin according to TCM standards [19].
4. Patients must be aged 18–75 years.
5. Patients must have a New York Heart Association cardiac functional class I–II
6. Patients are informed about the trial and voluntarily sign the consent form.

#### Exclusion criteria

1. Renal insufficiency, male serum creatinine >2.5 mg/dl (>220 μmol/L), female serum creatinine >2.0 mg/dl (>175 μmol/L)
2. Serious liver disease or alanine transaminase and aspartate transaminase values two times higher than the upper reference limit value
3. Systolic blood pressure >160 mmHg or diastolic blood pressure >100 mmHg
4. Presence of diabetes with random blood glucose level ≥13.7 mmol/L or HbA1c ≥9.5 %
5. Pregnant women or women who are preparing for pregnancy, and subjects who are known to be allergic to ingredients of the study drug
6. Acute cerebrovascular disease
7. Malignancy or having a life expectancy less than 3 years
8. Severe hematopoietic diseases
9. Severe psychiatric conditions
10. Previous involvement in other clinical trials or participation in other clinical trials within the past 3 months

### Withdrawal, dropout, and discontinuation

Participants can voluntarily withdraw at any time during the trial. Subjects who meet the inclusion criteria, fill out and sign the consent form, and successfully enter the randomized trial who fail to complete the observational period proposed in the trial, regardless of time and reasons, are considered as dropout cases. Reasons for withdrawal will be recorded in case report forms (CRFs), and the last data recorded for these participants will be included in the data analysis.

The trial can be terminated in the following circumstances: (1) occurrence of serious adverse events (AEs) related to the research medication, (2) the test drug is found to have no clinical value during the trial, (3) financial and management reasons, and/or (4) administrative authorities terminate the trial.

### Intervention

Eligible patients will be allocated to receive Xinyue capsule or placebo for 6 months in addition to conventional treatment, including antiplatelet, lipid-lowering, antihypertensive, or antidiabetic therapy, according to the clinical guidelines. The Xinyue capsules and placebo capsules (0.6 g ante cibum, three times daily for 24 weeks; China State Food and Drug Administration approval number Z20030073) will be provided by Jilin Jilin Jian Yisheng Pharmaceutical Co. Ltd. (Ji’An City, China). Each capsule is 0.3 g, equivalent to 50 mg of *P. quinquefolium* saponin. The Xinyue and placebo capsules have the same outer packaging, color, shape, and flavor.

### Outcomes and measures

The primary outcome is the time from randomization to the first occurrence of the major adverse cardiovascular event (MACE): cardiac death (any death unless an unequivocal noncardiac cause could be established), nonfatal MI (appearance of pathological Q waves that were absent at baseline or a total creatine kinase [CK] level more than two times the upper limit of normal [ULN] with presence of CK isoenzyme MB higher than the ULN), and urgent revascularization with either PCI or coronary artery bypass graft. The secondary outcome measures is the time from randomization to the first occurrence of, stroke, pulmonary embolism, and peripheral vascular events, as well as death due to any cause. Safety outcomes, which include a complete blood count, kidney and liver function tests, high-sensitivity C-reactive protein level, cardiac function index, blood lipid levels, Quality of Life Questionnaire (QLQ) score, 36-item Short Form Health Survey (SF-36) score, and TCM syndrome score will also be monitored periodically. All AEs will be followed from randomization to the end of the trial. Items to be measured and the time window of data collection are shown in Table 1.

**Table 1:** Measurement items and points of data capture

	Screening	Treatment period			Follow-up period	
	Visit 1	Run-in period (visit 2)	12 weeks (visit 3)	24 weeks (visit 4)	12 weeks (visit 5)	24 weeks (visit 6)
Informed consent	X					
Inclusion/exclusion criteria		X				
Demographic data	X					
Medical history	X					
Concomitant medications	X					
Tongue and pulse condition in TCM	X	X	X	X	X	X
Vital signs	X	X	X	X	X	X
12-lead ECG	X			X		X
Complete blood count, urine and stool tests	X			X		X
Liver and renal function tests	X			X		X
Coagulation function test	X			X		X
Adverse event evaluation			X	X	X	X
Dispense drug		X	X	X	X	X
Primary endpoint			X	X	X	X
Secondary endpoint			X	X	X	X
Cardiac function	X			X		X
hs-CRP	X			X		X
Lipid panels	X			X		X
QLR score	X		X	X	X	X
SF-36 score	X		X	X	X	X
TCM syndrome score	X		X	X	X	X

*Abbreviations: ECG* electrocardiogram, *hs-CRP* high-sensitivity C-reactive protein, *SF36* 36-item Short Form Health Survey, *TCM* traditional Chinese medicine, *QLQ* quality of Life Questionnaire​

X represents the indicators tested in the specific time period

Coagulation function test: prothrombin time, activated partial thromboplastin time, fibrinogen, thrombin time

Lipid panels: high-density lipoprotein, low-density lipoprotein, cholesterol, triglycerides

Primary endpoint: time from randomization to the first occurrence of the major adverse cardiovascular event

Secondary endpoint: time from randomization to the first occurrence of stroke, pulmonary embolism, and peripheral vascular events, as well as death due to any cause

### Adverse events

AEs are defined as negative or unintended clinical manifestations following the treatment. Patients will be asked to report to the investigators any abnormal reactions occurring at any time during the trial. In addition, investigators will collect information about abnormal reactions monthly. All details of related and unexpected AEs, such as time of occurrence, degree of AE, and suspected causes, will be recorded on CRFs. There is also a data safety monitoring board to oversee the trial.

### Study-specific visits and procedures

The schedule for all study-related procedures for all evaluations is shown in Table 1. For each procedure, subjects are to be assessed by the same investigator or site personnel whenever possible. The timing of each visit is relative to randomization (day 1). Baseline measures include demographic characteristics, medical history, medications, measurement of vital signs (temperature, blood pressure, breathing and heart rates), complete blood count, routine urine test, stool test, liver and kidney function tests, 12-lead electrocardiogram, cardiac ultrasound, TCM syndrome, and QLQ and SF-36 scores (details in the ​Additional file 2). All baseline measurements excluding the medical history will be repeated with all participants at visit 4 and visit 6. The TCM syndrome, QLQ, and SF-36 data will be recorded at visit 3 and visit 5. AEs and outcome measurements will be recorded from visit 3 to visit 6.

### Randomization and blinding

Participants are randomized in a 1:1 ratio using a computer-generated, site-stratified, block randomization schedule. The study capsule will be labeled with sequential randomization numbers, and each patient will be assigned the lowest number available at each participating center. All patients, care providers, and attending physicians will be blinded to treatment assignment until the study is completed. The duplicated blinding codes will be given to the main research institution and the manufacturer to keep, and the blinding codes cannot be broken during the trial.

### Date entry and quality control of data

CRFs have been used for data entry, and data from all participating centers will be imported into the clinical data management system (http://www.xyedc.com/). To maintain the quality of the data, we will adopt valid measures to ensure information accuracy, integrity, and authenticity. First, computer logic checks will be run to identify items such as inconsistent dates, missing data, and questionable values. After that, the supervisor will perform source data verification to check the consistency of the original data. Queries may be issued by the supervisor and will be answered by the site investigators (see in the additional file 3). Second, manual checks will identify more complicated and less common errors. Third, the supervisor will conduct the site visit to compare the electronic database with the source documents. Identified errors will be solved to ensure the data quality. Fourth, the Data Coordination Center will be in charge of data validation.

### Sample size calculation

The sample size was calculated on the basis of expected reduction in cardiovascular events (cardiac death, all-cause mortality, reoccurrence of MI, and any form of revascularization). A previous study suggested that the incidence for all major clinical cardiovascular events combined at 1 year after interventional treatment of CHD is 12.8 % [20]. Therefore, the hypothesis of this study is to reduce the incidence of cardiovascular events to 7 % in the treatment group. Given a type I error rate of α = 0.05, a power of 80 % (type II error rate of β = 0.2), the sample size for one arm needs to be 447, resulting in *n* = 2 × 447 = 894 patients. Considering a dropout rate of 20 %, a total of 1097 patients needs to be allocated to reach the required number of patients for the efficacy analysis. For convenience of randomization, we decided to recruit 1100 patients. The formula used to calculate the sample size is as follows:

$$ n=\frac{p_1\left(1-{p}_1\right)\kern0.5em +\kern0.5em {p}_2\left(1-{p}_2\right)}{{\left({p}_1-{p}_2\right)}^2}{\left(u{}_{\upalpha}+\kern0.5em u{}_{\upbeta}\right)}^2 $$

$$ \mathrm{n}\approx 447\ \mathrm{patients}/\ \mathrm{group} $$

### Statistical analysis

The data from all participating centers will be combined for statistical analysis of the primary and secondary endpoints as well as AEs. The analysis will be done at Beijing Jiaotong University. Continuous variables will be presented as the mean ± SD. The comparability of the characteristics between the two study groups will be assessed using a two-samples Student’s *t* test for continuous variables and the χ^2^ test or Wilcoxon test, when appropriate, for categorical variables. The Wilcoxon paired signed-rank test will be used for within-group comparisons.

All randomized patients constituted the intention-to-treat population. All participants will be analyzed according to their original treatment allocation. Primary and secondary endpoint data will be collected for the entire follow-up period for all patients. Patients lost to follow-up will be considered at risk until the date of last contact, at which point they will be censored. Kaplan-Meier curves will be used to examine MACE-free survival time. The Cox proportional hazards model with covariates of treatment will be used for primary and secondary analyses. Hazard ratios and 95 % confidence intervals for each of the categories determined by age, sex, diabetes, and conventional therapy will be provided for the primary and secondary endpoints.

For all analyses, a value of *P* < 0.05 will be considered statistically significant, and all tests will be two-tailed. All analyses will be conducted using SAS software version 9.2 (SAS Institute, Cary, NC, USA).

## Discussion

In this trial, we will investigate whether the Xinyue capsule combined with conventional treatment reduces the incidence of MACE and improve quality of life among patients within 1 year after PCI. With the pleiotropic effects that encompass antioxidant, antiapoptosis, and improvement of insulin resistance, PQS was demonstrated to modify regional endothelial function, decrease oxidative stress and blood glucose, and induce angiogenesis [21–24]. In our previous studies, we have found that Xinyue capsules plus Chuanxiong capsules combined with conventional treatment can further reduce the incidence of cardiovascular events without any adverse effect in patients with ACS after PCI [16]. A small-sample clinical trial in which researchers recruited 100 patients with ACS after successful PCI showed that Xinyue capsules together with Western medicine treatment can improve cardiac function and quality of life [25]. These findings and observations serve as an impetus for large controlled trials to examine the effect of Xinyue capsules in patients with ACS after successful PCI. The present study is designed as a multicenter, double-blind, randomized, placebo-controlled, parallel-group superiority trial that will provide higher-powered evidence regarding long-term survival with the use of Xinyue capsules in addition to conventional treatment for patients with CHD after successful PCI.

Our study has some limitations. First, the study is being undertaken in China, so it is uncertain whether the relative effects of the trial drugs would be similar in other ethnic groups. The relatively short follow-up period is another limitation of the study.

To conclude, the aim of this trial is to demonstrate that Xinyue capsules plus conventional treatment will lead to a reduction in the incidence of MACE in patients with CHD after successful PCI and subsequently yield long-term benefit.

### Trial status

The trial was initiated in December 2014 and is currently open for enrollment. A total of 452 participants have been enrolled. However, no analysis has been conducted since the commencement of the trial.

## Abbreviations

ACS, acute coronary syndrome; AE, adverse event; CHD, coronary heart disease; CK, creatine kinase; CRF, case report form; ECG, electrocardiogram; hs-CRP, high-sensitivity C-reactive protein; MACE, major adverse cardiovascular event; MI, myocardial infarction; PCI, percutaneous coronary intervention; PQS, *Panax quinquefolius* saponin; QLQ, Quality of Life Questionnaire; SF-36, 36-item Short Form Health Survey; TCM, traditional Chinese medicine; ULN, upper limit of normal

## Additional files

Additional file 1: Institutional review board of 25 participating centers. (PDF 82 kb) Additional file 2: Case report form 2014.3.4R2. (PDF 1119 kb) Additional file 3: Investigators brochure R2. (PDF 943 kb)
