# Supplementary material for: Effect of Xinyue capsules on patients with coronary heart disease after percutaneous coronary intervention: study protocol for a randomized controlled trial
Source: Trials. 2016 Aug 18;17:412. doi: 10.1186/s13063-016-1531-x (PMC4991005; doi:10.1186/s13063-016-1531-x)
Supplement: Additional file 1: — Institutional review board of 25 participating centers. (PDF 82 kb) [file 13063_2016_1531_MOESM1_ESM.pdf]

Institutional review board of 25 participating centers

1. Xiyuan Hospital of China Academy of Chinese Medical Sciences;
2. Beijing An Zhen Hospital of the Capital University of Medical Sciences;
3. Second Artillery General Hospital of people's Liberation Army China;
4. Dongzhimen Hospital, Beijing University of Chinese Medicine;
5. Navy General Hospital of PLA,
6. Beijing Hospital of Capital Medical University;
7. Xia men hospital of TCM;
8. Haici Hospital of Medical College of Qiingdao University;
9. The First Hospital of Changchun University of TCM;
10. The 309th hospital of PLA
11. The first people's Hospital of Yunnan Province
12. GuangDong Province Hospital Of Traditional Chinese Medicine,
13. Luoyang Central Hospital
14. The hosptial of TCM of Xinjiang Uygur Autonomous Region
15. The hospital of Gansu College of Traditional Chinese Medicine
16. Wuxi Hospital of traditional Chinese Medicine
17. Hebei Cangzhou Hospital of integrated traditional Chinese and Western Medicine
18. The First Hospital of Henan University of Traditional Chinese Medicine,
19. Henan Anyang People's Hospital,
20. The First Hospital of Hunan University of Chinese Medicine
21. Xuzhou Hospital of Traditional Chinese Medicine,
22. Shuguang Hospital of Shanghai University of Traditional Chinese Medicine,
23. The Hospital of Medical College of Qiingdao University
24. the Hospital of Guiyang Medical College.
25. The First Hospital of Yanbian University
