# Supplementary material for: Effect of Xinyue capsules on patients with coronary heart disease after percutaneous coronary intervention: study protocol for a randomized controlled trial
Source: Trials. 2016 Aug 18;17:412. doi: 10.1186/s13063-016-1531-x (PMC4991005; doi:10.1186/s13063-016-1531-x)
Supplement: Additional file 2: — Case report form 2014.3.4R2. (PDF 1119 kb) [file 13063_2016_1531_MOESM2_ESM.pdf]

项目编号：11ZDZH005

吉林省重大科技成果转化项目——
创新药物“心悦胶囊”产业化技术开发

心悦胶囊治疗介入后冠心病的随机、双盲、安慰剂对照临床试验

研究病历
（Case Report Form）

患者姓名拼音缩写：□□□□

患者姓名：______________________

研究单位：______________________

研究者姓名：____________________

入组时间：______________________

中国中医科学院西苑医院

<u>在正式填表前，请认真阅读下列填表说明</u>

病例报告表填写说明

1. 筛选合格者填写本观察表。
2. 病例调查表应用签字笔用力填写，不得用铅笔或圆珠笔填写。在填写过程中，请将垫板垫在每页两联单下面，以避免此页笔迹印在下页。
3. 表中凡有“□”的项，请在符合的条目上划“√” 。
4. <u>所有检查项目及表格均须填写，不得缺项、漏项</u>；具体用药剂量和时间不明，请填写“UK” 。
5. 观察表的每页均须填写医院编号、随机号、患者姓名缩写、组别，观察医生必须签署姓名和日期。
6. 表格填写务必准确、清晰，任何数据禁止擦除或涂墨。如有错误发生，可在错误处上方书写正确值，将错误值用横线居中划出，修改者签名并加注日期，必要时说明理由。举例：~~58.6~~ 56.8 LW 11 03 31
7. 试验期间应如实填写合并用药记录表、不良事件记录表。如有严重不良事件发生，无论是否与所研究的药物有关，均需及时报告项目负责总单位，然后由项目负责单位上报。
8. 各实验室检查的检查报告、化验单，均应贴在表格的最后一面。
9. 患者姓名缩写四格需填满，两字姓名填写两字拼音前两个字母；三字姓名填写三字首字母及第三字第二字母；四字姓名填写每一个字的首字母。

举例：张红

Z	H	H	O

李叔明

L	S	M	I

欧阳小惠

O	Y	X	H

心悦胶囊治疗介入后冠心病的随机、双盲、安慰剂对照临床研究
知情同意书

欢迎您自愿参加吉林省科委创新药物成果转化项目“心悦胶囊治疗介入后冠心病的随机、双盲、安慰剂对照临床研究”，并对您的参加表示衷心的感谢！在您决定是否参加该项研究之前，您有必要了解本研究的目的、试验药品、可能给您带来的风险、本研究需要您配合的事情以及您作为受试者的权利，请您仔细阅读这份受试者须知。

1．药物简介

心悦胶囊为国家上市5类新药，其主要成分为西洋参茎叶总皂苷，具有益气养阴、活血化瘀之功效，主要用于冠心病所引起的胸闷胸痛、憋气、心悸气短等症。毒理实验未发现明显的毒副反应，前期临床试验未发现严重不良反应。

本研究目的是在西医常规治疗的基础上，按照循证医学方法，观察心悦胶囊对介入术后冠心病患者的终点事件和心功能改善情况，进一步证实心悦胶囊降低介入后冠心病的终点事件发生率和改善心功能的客观疗效。

2．法规背景

本课题为吉林省科学技术委员会创新药物成果转化项目，并获得中国中医科学院西苑医院伦理委员会批准。

3．总体过程

（1）入选研究前，将进行以下检查以确定您是否可以参加研究。医生将询问、记录您的病史、对您进行体格检查。您需要做血常规、尿常规、大便常规、肝功能、肾功能、心电图、心脏超声等理化检查，若检查结果符合入组标准，您将根据研究要求进行治疗。

（2）研究开始将根据随机方法，决定您接受西医常规治疗或西医常规结合中药治疗，您和您的医生都无法事先知道和选择任何一种治疗方法。

（3）开始临床观察后，您应该按照项目规定的时间就诊，并如实向医生反映病情变化，医生将收集您的病史、体检结果及相关理化检测结果。

（4）要您配合的其他事项：

- ✓ 您必须按照医生和您约定的随访时间来就诊。您的随访非常重要，因为医生将根据您的具体情况决定您是否继续参加该项试验研究。
- ✓ 您必须按医生指导用药，并请您在每次用药后及时、客观地在用药记录卡中记录，同时要记录试验期间您由于其它疾病必须继续服用的药物。
- ✓ 在试验期间除本研究方案中规定的用药外，您不能随意使用与本病治疗相关的其它药物。如您需要其它治疗，请事先与您的医生联系。

4．您的权利和利益

⑴ 研究人员将向您介绍研究中所用药品及研究安排，参加与否完全遵循自愿的原则。研究者向您报告所有与您有关的事件，以便您随时决定是否继续参加。如果您有任何问题，您可打电话或直接向研究者咨询。您将在研究期间获得良好的医疗服务。您可以拒绝参加此项研究，或在研究过程中的任何时间退出研究，这都不会影响您和医生的关系，都不会影响对您的医疗或有其他方面利益的损失。

如果您不参加本项研究，或中途退出，还有很多其它替代的治疗药物。您不必为了您的疾病而必须选择参加本项研究。

⑵ 您在本项试验中的受益。此种受益包括您的病情可能获得改善，根据您的证型您将获得半年免费使用中成药治疗的机会。研究过程中，您将会得到参与单位（三甲医院）心血管科的免费健康体检和随访观察，并随时提供医疗服务。

⑶ 如果你是被分配在西医常规治疗组，你将严格按照目前西医规范治疗。

⑷ 如果您同时合并其他疾病需要中药治疗，将不在免费范围之内。

5．您的义务

⑴ 坚持自愿参加的原则，在试验开始之前签署知情同意书。

⑵ 遵循临床试验方案，听从研究者统一安排。配合研究者完成试验任务。

⑶ 试验期间不得擅自使用其他药物，如必须使用请在研究者指导下应用。

6．可能出现的不良反应和安全措施

所有治疗药物都有可能产生副作用。如果在研究中您出现任何不适，或病情发生新的变化，或任何意外情况，不管是否与药物有关，均应及时通知您的医生，他/她将对此做出判断和医疗处理，医生和研究者将尽全力预防由于本研究可能带来的伤害。如果在临床试验中出现不良事件，医学专家委员会鉴定其是否与试验药物有关。申办者将根据相关文件规定，对试验相关的损害提供治疗的费用及相应的经济补偿。这一点已经在我国《药物临床试验质量管理规范》中做出了规定。您在研究期间需要按时随访，这些都可能给您造成麻烦或带来不方便。

7．终止您参加研究的理由

⑴ 研究者判定继续进行研究对您有害。

⑵ 您未按照医生的指导用药。

⑶ 研究过程中发生了严重不良反应。

⑷ 临床研究被取消。

发生了上述情况，研究者有权不征得您的同意而终止您参加研究。

8、保密性

只有授权的研究者才能查阅您的记录。报告中不会出现您的姓名和身份证明。同时我们允许国家中医药管理局主管部门审阅记录。

知情同意书签字页

临床研究项目名称：心悦胶囊治疗介入后冠心病的随机、双盲、安慰剂对照临床研究

课题组织单位：中国中医科学院西苑医院

课题编号：11ZDZH005

协作单位：北京中医药大学附属东直门医院、甘肃中医学院附属医院、广东省深圳市第三人民医院、广州省中医院、贵阳医学院附属医院、海军总医院、河北沧州中西医结合医院、河南安阳市人民医院、河南中医学院第一附属医院、湖南中医药大学第一附属医院、洛阳市中心医院、青岛大学医学院附属海慈医院、青岛大学医学院附属医院、厦门市中医院、首都医科大学附属北京安贞医院、首都医科大学附属北京中医院、泰安市中医院、无锡市中医院、新疆维吾尔自治区中医院、徐州市中医院、延边大学附属第一医院、长春中医药大学第一附属医院、中国医科大学附属阜外心血管病医院、中日友好医院（按照拼音顺序排列）

同意声明

我已经阅读了上述有关本研究的介绍，而且有机会就此项研究与医生讨论并提出问题，我提出的所有问题都得到了满意的答复。我知道参加本研究可能产生的风险和受益。我知晓参加研究是自愿的，我确认已有充足时间对此进行考虑，而且明白：

- 本研究的目的和意义；
- 本研究的方法和程序；
- 本项研究的利和弊；
- 受试者的权利和利益；
- 受试者的义务和需要配合研究者做哪些工作；
- 统计资料中不使用受试者全名的意义；
- 完全自愿参加并可随时退出的原则。

我已阅读了受试者须知，同意作为志愿受试者参加本项目研究，并愿意与研究者配合，共同完成本项研究任务。

受试者签名：__________________ 日期： _______年_______月_______日

联系电话：____________________

研究者签名：__________________ 日期： ________年_______月_______日

联系电话：____________________

心悦胶囊治疗介入后冠心病的临床研究流程图

研究阶段		导入期（7天）	治　疗　期（用药期）			随访期（用药后）	
			治疗开始	第12周	第24周	第12周	第24周
签署知情同意书		√					
入选/排除标准		√					
问诊	一般情况	√					
	现病史	√					
	既往病史	√					
	合并用药情况	√					
查体	舌、脉	√	√	√	√	√	√
	生命体征	√	√	√	√	√	√
	体格检查	√	√	√	√	√	√
安全性观察	静息心电图		√		√		√
	血尿便常规		√		√		√
	肝功、肾功		√		√		√
	凝血功能*		√		√		√
	不良事件		√	√	√	√	√
药物发放			√	√	√		
疗效观察	主要终点指标		√	√	√	√	√
	次要终点指标		√	√	√	√	√
	心功能指标**		√		√		√
	运动耐量***		√	√	√		
	炎症指标		√		√		√
	血脂指标****		√		√		√
	自评量表		√		√		√
	中医症状评分		√		√		√

注：*凝血功能包括凝血酶原时间、活化部分凝血活酶时间、纤维蛋白原和凝血酶时间；**心功能指标包括心脏彩超（左室舒张末期容积、右室舒张末期容积、射血分数）和pro-BNP；　***运动耐量指标主要包括平板运动耐量和诱发缺血时间（仅前100例患者），分别于服药开始、服药12周、服药24周进行；****血脂检查包括血浆总胆固醇（TC）、甘油三酯（TG）、低密度脂蛋白胆固醇（LDL-C）、高密度脂蛋白胆固醇（HDL-C）。

病例纳入标准

提示："纳入标准"中若有任一项为"否"，则病人不能入选。

入选标准		是 1	否 0
1	符合冠心病诊断，并成功行冠脉介入治疗者；	□	□
2	介入治疗后 3-12 个月病情稳定者：①无进展性胸痛；②肌钙蛋白正常或轻度升高，但低于高值上线 99 百分数；③心绞痛Ⅰ-Ⅱ级（CCS 分级）；	□	□
3	中医辨证为气虚或气阴两虚证的患者；	□	□
4	年龄≤75 岁；	□	□
5	心功能Ⅰ-Ⅱ级(NYHA 心功能分级)；	□	□
6	试者知情，自愿签署知情同意书。	□	□

病例排除标准

提示："排除标准"中若有任一项为"是"，则病人不能入选。

排除标准		是 1	否 0
1	肾功能不全，男性血清肌酐>2.5 mg/dl（>220umo/l）女性>2.0 mg/（>175umo/l）；	□	□
2	患有明显的肝脏疾患或 ALT、AST 高于正常上限 3 倍；	□	□
3	控制后收缩压>160mmHg 或舒张压>100mmHg（测量血压前患者至少需要静坐 5 分钟）；	□	□
4	控制后随机血糖≥13.7mmol/L 的糖尿病患者或糖化血红蛋白≥9.5%；	□	□
5	妊娠或准备妊娠妇女，哺乳期妇女及对研究药物已知成分过敏者；	□	□
6	合并急性脑血管疾病患者；	□	□
7	恶性肿瘤或患者预期寿命少于 3 年；	□	□
8	严重的造血系统疾病患者；	□	□
9	严重的精神病患者；	□	□
10	近三个月内参加过或正在参加其它临床试验者。	□	□

心悦胶囊治疗介入后冠心病的临床研究病例观察表

病例观察记录表(一)（导入期）

病人基本信息

门诊号：□□□□□□ 住院号：□□□□□□□

性别：□ （男为1，女为2） 年龄□□岁 身高：□□□cm

体重：□□□kg 病程：□□（年□，月□，填以最接近的约数）

联系电话1：______________联系人：______________

联系电话2：______________联系人：______________

联系地址：____

嗜好：吸烟 □无；□有 ____支/日，____年，已戒____年

饮酒 □无；□有 ____年, 饮酒种类 □白酒 □啤酒 □红酒

饮酒量 □□□ml/次 饮酒频率 □□次/周

查体： 心率：□□□次/分 心律：□齐 □不齐 血压：□□□/□□□mmHg

有无出血点或瘀斑 □无； □有 __________________

其它阳性体征 □无； □有 __________________

合并病及用药 目前合用的西药 无 □ 有 □

诊 断	无	有	分型	病史	合并用药	剂量	合并用药	剂量
糖尿病	□	□	□型	□□年	□磺酰脲类		□α -糖苷酶抑制剂	
					□双胍类		□促胰岛素分泌药	
					□胰岛素增敏剂		□胰岛素	
高血压	□	□	□级	□□年	□利尿剂		□β 受体阻滞剂	
					□钙通道阻滞剂		□ACEI 类	
					□ARB 类		□其他	
血脂异常	□	□		□□年	□他汀类		□贝特类	
					□烟酸		□其他	
冠心病	□	□		□□年	□阿司匹林		□氯吡格雷	
					□其他		□其他	
其他	□	□		□□年				

医院编号	随机号	受试者姓名拼音缩写	组　别
□□□	□□□□	□□□□	□ A组　□ B组

冠状动脉造影结果：（_____年____月____日）

冠脉病变位置△	支架术前狭窄程度		支架名称▲		支架型号	支架术后残余狭窄	
	无	有	代号	其他		无	有
	□	□□□%	□	□	□□mm*□□cm	□	□□□%
	□	□□□%	□	□	□□mm*□□cm	□	□□□%
	□	□□□%	□	□	□□mm*□□cm	□	□□□%
	□	□□□%	□	□	□□mm*□□cm	□	□□□%
	□	□□□%	□	□	□□mm*□□cm	□	□□□%
	□	□□□%	□	□	□□mm*□□cm	□	□□□%
	□	□□□%	□	□	□□mm*□□cm	□	□□□%
	□	□□□%	□	□	□□mm*□□cm	□	□□□%

△注：将冠状动脉分段图中病变位置的代号填入表格中

▲注:1 firebird; 2 endeavor; 3 cypher; 4 partner ; 5 taxus; 6 excel; 7 coroflex; 8 xience V

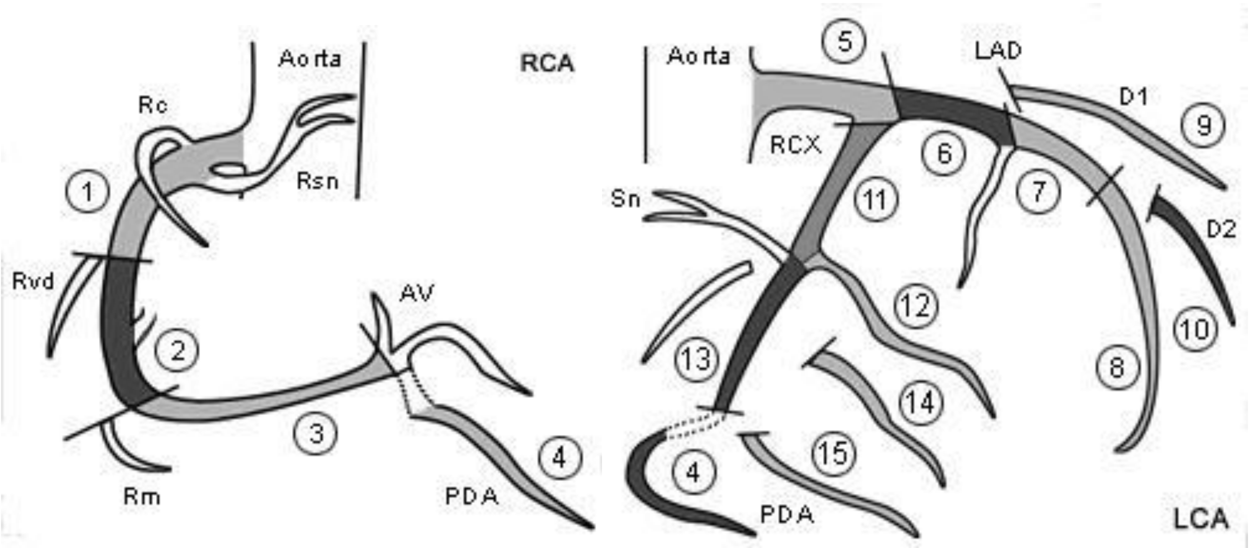

医院编号	随机号	受试者姓名拼音缩写	组　别
□□□	□□□□	□□□□	□ A组　□ B组

中医症状积分（包括中医兼证）

中医症状	无	轻　度（1分）	中　度（2分）	重　度（3分）
形体肥胖	□#	□体重指数＞25	□□体重指数＞30	□□体重指数＞35
气短	□	□日常活动，感气短	□轻微活动，感气短	□不活动，感气短
乏力	□	□日常活动，感乏力	□轻微活动，感乏力	□不活动，感乏力
五心烦热	□			
盗汗	□			
口干不欲饮	□	□口干，可忍受	□口干，常需漱口	□ 持续口干，夜间尤甚
固定性刺痛	□	□刺痛轻微，可自行缓解	□刺痛明显，需含药缓解	□刺痛重，持续不缓解
口唇紫暗	□	□口唇稍暗	□口唇紫暗	□口唇紫暗发黑
爪甲紫暗	□	□爪甲稍暗	□爪甲紫暗	□爪甲紫暗发黑
肢体沉重	□	□活动时，觉肢体沉重	□轻微活动，感肢体沉重	□不活动，感肢体沉重
四肢困倦	□	□困倦，不影响工作	□困倦，影响工作	□困倦，严重影响工作
口中粘腻	□	□口中粘腻，稍有不爽	□口中粘腻，严重不爽	□口中粘腻，偶有痰涎
其他	□	□	□	□
其他	□	□	□	□

#体重指数≤25

证候分级：轻□ 中□ 重□。分级标准：轻：总分≤11分；中：总分12-21分；重：总分≥22分。

舌脉情况

项目	具体征象
舌面	□正常 □胖大 □瘦小 □老 □嫩 □淡 □紫 □暗 □红 □瘀斑 □瘀点 □齿痕<br>□其他：________
舌下静脉丛	□迂曲 □怒张 □紫红 □绛紫 □其他：________
舌苔	□ 正常 □厚 □薄 □少 □剥 □无 □白 □黄 □腻 □少津<br>□ 其他：________
脉象	□ 正常 □沉 □迟 □数 □细 □弦 □弱 □滑 □涩 □促 □结 □代<br>□ 其他：________

病例观察记录表(二)（治疗开始）

查体： 心率：□□□次/分 心律：□齐 □不齐______________________________

血压：□□□/□□□mmHg

有无出血点或瘀斑 □无； □有 ___________________

其它阳性体征 □无； □有 ___________________

中医症状积分（包括中医兼证）

中医症状	无	轻 度（1分）	中 度（2分）	重 度（3分）
形体肥胖	□#	□体重指数＞25	□□体重指数＞30	□□体重指数＞35
胸闷	□	□胸闷轻微	□明显，有时叹息	□胸闷如窒，叹息不止
固定性刺痛	□	□刺痛轻微，可自行缓解	□刺痛明显，需含药缓解	□刺痛重，持续不缓解
气短	□	□日常活动，感气短	□轻微活动，感气短	□不活动，感气短
乏力	□	□日常活动，感乏力	□轻微活动，感乏力	□不活动，感乏力
口干不欲饮	□	□口干，可忍受	□口干，常需漱口	□ 持续口干，夜间尤甚
口唇紫暗	□	□口唇稍暗	□口唇紫暗	□口唇紫暗发黑
爪甲紫暗	□	□爪甲稍暗	□爪甲紫暗	□爪甲紫暗发黑
肢体沉重	□	□活动时，觉肢体沉重	□轻微活动，感肢体沉重	□不活动，感肢体沉重
四肢困倦	□	□困倦，不影响工作	□困倦，影响工作	□困倦，严重影响工作
口中粘腻	□	□口中粘腻，稍有不爽	□口中粘腻，严重不爽	□口中粘腻，偶有痰涎
其他	□	□	□	□
其他	□	□	□	□
其他	□	□	□	□

#体重指数≤25

证候分级：轻□ 中□ 重□。分级标准：轻：总分≤11分；中：总分12-21分；重：总分≥22分。

生活质量自评量表-简明健康调查问卷(SF-36)

下面的问题是询问您对自己健康状况的看法、您的感觉如何、以及您进行日常活动的能力如何，每个问题请选择一个最适合您的答案，并在答案后的“○”中打“√”。

1.总体来讲，您的健康状况是：

	请打一个钩
非常好	○
很好	○
好	○
一般	○
差	○

2.跟一年前相比，您觉得您现在的健康状况是：

	请打一个钩
比一年前好多了	○
比一年前好一些	○
和一年前差不多	○
比一年前差一些	○
比一年前差多了	○

健康和日常活动

3. 以下这些活动都与日常活动有关，您的健康状况是否限制了这些活动？如果有限制，程度如何？

	有很多限制	*有一定限制*	*根本没限制*
（1）重体力活动（跑步、举重、激烈运动）	○	○	○
（2）适度活动（如移桌子、扫地、做操）	○	○	○
（3）手提或搬运日常用品（如买菜、购物）	○	○	○
（4）上几层楼梯	○	○	○
（5）上一层楼梯	○	○	○
（6）弯腰、屈膝、下蹲	○	○	○
（7）步行 1500 米左右的路程	○	○	○
（8）步行 800 米左右的路程	○	○	○
（9）步行约 100 米的路程	○	○	○
（10）自己洗澡、穿衣	○	○	○

4. 在过去四个星期里，您的工作和日常活动有没有因为身体的原因而出现以下这些问题？

每个问题都回答有或没	有	没有
（1）减少了工作或其他活动的时间	○	○
（2）本来想要做的事情减少了一部分	○	○
（3）想要做的工作或活动的种类受到限制	○	○
（4）完成工作或其他活动有困难（比如需要额外的努力）	○	○

5. 在过去四个星期里，您的工作和日常活动有没有因为情绪（如感到消沉或忧虑）的原因而出现以下这些问题？

每个问题都回答有或没	有	没有
（1）减少了工作或其他活动的时间	○	○
（2）本来想要做的事情只能完成一部分	○	○
（3）做工作或其他活动不如平常仔细	○	○

6. 在过去四个星期里，您的身体健康和情绪不好在多大程度上影响了家人朋友、邻居或集体的正常社交活动？

请打一个钩	
根本没有影响	○
很少有影响	○
中度有影响	○
有较大影响	○
有极大影响	○

7. 在过去四个星期里，您有身体上疼痛吗？

请打一个钩	
根本没有疼痛	○
有很轻微疼痛	○
有轻微疼痛	○
有中度疼痛	○
有严重疼痛	○
有很严重疼痛	○

8. 在过去四个星期里，身体上的疼痛影响了您的正常工作了吗（包括上班工作和家务活）？

请打一个钩

根本没有影响	○
很少有影响	○
中度有影响	○
有较大影响	○
有极大影响	○

您的感觉

9. 以下这些问题有关过去一个月里您的感觉如何以及您的情况如何。（对每一条问题，请钩出最接近您的感觉的那个答案）

请在每一行打一个钩

在过去一个月里持续的时间	所有的时间	大部分时间	比较多时间	一部分时间	没有此感觉
（1）您觉得生活充实吗？	○	○	○	○	○
（2）您是一个精神紧张的人吗？	○	○	○	○	○
（3）您感到垂头丧气，什么事都不能使你振作起来吗？	○	○	○	○	○
（4）您觉得心情平静吗？	○	○	○	○	○
（5）您精力充沛吗？	○	○	○	○	○
（6）您的情绪低落吗？	○	○	○	○	○
（7）您觉得筋疲力尽吗？	○	○	○	○	○
（8）您是个快乐的人吗？	○	○	○	○	○
（9）您感觉疲劳吗？	○	○	○	○	○

10. 过去的四周内，您的健康或情绪问题是否影响了您的社交活动（比如走亲访友）？

请打一个钩

所有的时间	○
大部分时间	○
比较多时间	○
一部分时间	○
根本没有	○

总的健康情况

11. 请对下面的每一句话，选出最符合您情况的答案

请在每一行打一个钩

	绝对正确	大部分正确	不能肯定	一部分错误	绝对错误
（1）我好像比别人容易生病	○	○	○	○	○
（2）我跟我认识的人一样健康	○	○	○	○	○
（3）我认为我的健康状况在变坏	○	○	○	○	○
（4）我的健康状况非常好	○	○	○	○	○

舌脉情况

项目	具体征象
舌面	□正常 □胖大 □瘦小 □老 □嫩 □淡 □紫 □暗 □红 □瘀斑 □瘀点 □齿痕<br>□其他：________
舌下静脉丛	□迂曲 □怒张 □紫红 □绛紫 □其他：________
舌苔	□ 正常 □厚 □薄 □少 □剥 □无 □白 □黄 □腻 □少津 其他：________
脉象	□ 正常 □沉 □迟 □数 □细 □弦 □弱 □滑 □涩 □促 □结 □代<br>□ 其他：________

理化检查(_____年__月__日)：

检测项目		结 果	指标单位	检测项目	结 果	指标单位
血常规	RBC		×10^{12}/L	Hb		g/L
	WBC		×10^{9}/L	PLT		×10^{9}/L
	PDW		fL	N		%
	L		%			
尿常规	白细胞		个/HP	红细胞		个/HP
	蛋白质		g/L	葡萄糖		mmol/L
便常规	白细胞		个/HP	红细胞		个/HP
	便潜血		定性			
生化指标	ALT		U/L	AST		U/L
	BUN		mmol/L	Cr		μmol/L
	GRF		%	TC		mmol/L
	TG		mmol/L	LDL-C		mmol/L
	GLU		mmol/L	HDL-C		mmol/L
凝血四项	PT		s	APTT		s
	Fbg		g/L	TT		s
心功能	BNP		ng/l	Pro-BNP		ng/l
炎症	CRP		mg/l	高敏 CRP		mg/l

医院编号	随机号	受试者姓名拼音缩写	组 别
☐☐☐	☐☐☐☐	☐☐☐☐	☐ A 组 ☐ B 组

心电图活动平板运动试验检查记录（部分患者行此检查）

陈旧性心梗病史：☐0 无 ☐1 有	冠造或核素检查诊断依据 ☐0 无 ☐1 有
运动前心率： 次/分	运动试验终止的心率 分/次
**ST 段下降者填下表** ☐0 无 ☐1 有→填下表	
ST 段下降 0.1mV 的时间：____分____秒	下降最大的 ST 段恢复到运动前水平所需时间：___分___秒

运动全过程中心电图 ST 段下降最大幅度（mV）	I		II		III		aVR		aVL		aVF	
	V1		V2		V3		V4		V5		V6	
ST 段最大下降幅度和（mV）	Σ =											

**ST 段抬高者填下表** ☐0 无 ☐1 有→填下表	有陈旧心梗者表明梗塞部位：
心电图 ST 段开始抬高的时间：____分____秒	心电图 ST 段抬高最大幅度的持续时间：____分____秒

运动全过程中心电图 ST 段抬高最大幅度（mV）	I		II		III		aVR		aVL		aVF	
	V1		V2		V3		V4		V5		V6	
ST 段最大抬高幅度和（mV）	Σ =											

运动试验级别： ☐级	代谢当量（MET）：

运动试验全过程的血压监测	运动试验开始时的血压值 mmHg	
	全运动试验中血压上升的最高值（以收缩为标准） mmHg	
	血压上升到最高值所需时间 分/秒	
	**血压不升或下降者填写下表** ☐0 否 ☐1 是→填下表	
	收缩压上升<20mmHg 者的血压值 mmHg， 此时运动级别 ☐级	
	收缩压下降到最低时血压值 mmHg	下降到最低值时间___ 分___秒

运动试验终止的时间 ____ 分____秒	
运动试验终止的原因	心绞痛时使用硝酸甘油： ☐有 ☐无

心绞痛诱发因素 寒冷 ☐ 劳累 ☐ 情志 ☐ 其它 ☐

普通心电图检测纪录（无ST-T改变者划/）：

各导联ST段下降幅度(mV)	I		II		III		aVR		aVL		aVF	
	V1		V2		V3		V4		V5		V6	
ST 段下降幅度和（mV）	Σ =											
以波为主导联 T 波倒置幅度（mV）	I		II		III		aVR		aVL		aVF	
	V1		V2		V3		V4		V5		V6	
T 波幅度和（mV）	Σ =											

心电图(_____年____月____日)

心率：□□□次/分；基础心律：□窦性 □房颤 □室早 其他________________

□心律失常：□室早 □室上早 □室速 □室上速

□房室传导阻滞：□Ⅰ度 □Ⅱ度Ⅰ型 □Ⅱ度Ⅱ型 □Ⅲ度

□LBBB □RBBB □LAHB □LPHB

□其它________________

□ST 改变：

□下斜性下移：□Ⅰ□Ⅱ□Ⅲ□aVR□aVL□aVF□V1□V2□V3□V4□V5□V6，最大下移_____mV。

□水平性下移：□Ⅰ□Ⅱ□Ⅲ□aVR□aVL□aVF□V1□V2□V3□V4□V5□V6，最大下移_____mV。

□抬高：□Ⅰ□Ⅱ□Ⅲ□aVR□aVL□aVF□V1□V2□V3□V4□V5□V6 最大抬高_____mV。

□其它描述：____

超声心动图(_____年____月____日)：

□心内结构及血流未见异常

□异常：

□室壁运动异常

□室壁节段性或反向运动，部位：□前间壁 □前壁 □广泛前壁 □下壁

□侧壁 □高侧壁 □正后壁

□室壁运动消失，部位：□前间壁 □前壁 □广泛前壁 □下壁

□侧壁 □高侧壁 □正后壁

□室壁运动减弱，部位：□前间壁 □前壁 □广泛前壁 □下壁

□侧壁 □高侧壁 □正后壁

□瓣膜关闭不全或返流

□二尖瓣 □三尖瓣 □主动脉瓣 □肺动脉瓣

□其他描述：____

检测值：左室舒张末期内径：□□□mm；EF：□□%；

研究者：______________ 填表日期：_____年____月____日

用药前检验报告粘贴单

病例观察记录表(三)（治疗 12 周）

查体： 心率：□□□次/分　心律：□齐 □不齐______________________________

血压：□□□/□□□mmHg

有无出血点或瘀斑　□无；　□有 ____________________

其它阳性体征　□无；　□有 ____________________

中医症状积分（包括中医兼证）

中医症状	无	轻　度（1 分）	中　度（2 分）	重　度（3 分）
形体肥胖	□#	□体重指数＞25	□□体重指数＞30	□□体重指数＞35
胸闷	□	□胸闷轻微	□明显，有时叹息	□胸闷如窒，叹息不止
固定性刺痛	□	□刺痛轻微，可自行缓解	□刺痛明显，需含药缓解	□刺痛重，持续不缓解
气短	□	□日常活动，感气短	□轻微活动，感气短	□不活动，感气短
乏力	□	□日常活动，感乏力	□轻微活动，感乏力	□不活动，感乏力
口干不欲饮	□	□口干，可忍受	□口干，常需漱口	□ 持续口干，夜间尤甚
口唇紫暗	□	□口唇稍暗	□口唇紫暗	□口唇紫暗发黑
爪甲紫暗	□	□爪甲稍暗	□爪甲紫暗	□爪甲紫暗发黑
肢体沉重	□	□活动时，觉肢体沉重	□轻微活动，感肢体沉重	□不活动，感肢体沉重
四肢困倦	□	□困倦，不影响工作	□困倦，影响工作	□困倦，严重影响工作
口中粘腻	□	□口中粘腻，稍有不爽	□口中粘腻，严重不爽	□口中粘腻，偶有痰涎
其他	□	□	□	□
其他	□	□	□	□
其他	□	□	□	□

#体重指数≤25

证候分级：轻□ 中□ 重□。分级标准：轻：总分≤11 分；中：总分 12-21 分；重：总分≥22 分。

生活质量自评量表-简明健康调查问卷(SF-36)

下面的问题是询问您对自己健康状况的看法、您的感觉如何、以及您进行日常活动的能力如何，每个问题请选择一个最适合您的答案，并在答案后的“○”中打“√”。

1.总体来讲，您的健康状况是：

	请打一个钩
非常好	○
很好	○
好	○
一般	○
差	○

2.跟一年前相比，您觉得您现在的健康状况是：

	请打一个钩
比一年前好多了	○
比一年前好一些	○
和一年前差不多	○
比一年前差一些	○
比一年前差多了	○

健康和日常活动

3. 以下这些活动都与日常活动有关，您的健康状况是否限制了这些活动？如果有限制，程度如何？

	有很多限制	*有一定限制*	*根本没限制*
（1）重体力活动（跑步、举重、激烈运动）	○	○	○
（2）适度活动（如移桌子、扫地、做操）	○	○	○
（3）手提或搬运日常用品（如买菜、购物）	○	○	○
（4）上几层楼梯	○	○	○
（5）上一层楼梯	○	○	○
（6）弯腰、屈膝、下蹲	○	○	○
（7）步行 1500 米左右的路程	○	○	○
（8）步行 800 米左右的路程	○	○	○
（9）步行约 100 米的路程	○	○	○
（10）自己洗澡、穿衣	○	○	○

4.在过去四个星期里，您的工作和日常活动有没有因为身体的原因而出现以下这些问题？

每个问题都回答有或没

	有	没有
（1）减少了工作或其他活动的时间	○	○
（2）本来想要做的事情减少了一部分	○	○
（3）想要做的工作或活动的种类受到限制	○	○
（4）完成工作或其他活动有困难（比如需要额外的努力）	○	○

5. 在过去四个星期里，您的工作和日常活动有没有因为情绪（如感到消沉或忧虑）的原因而出现以下这些问题？

每个问题都回答有或没

	有	没有
（1）减少了工作或其他活动的时间	○	○
（2）本来想要做的事情只能完成一部分	○	○
（3）做工作或其他活动不如平常仔细	○	○

6. 在过去四个星期里，您的身体健康和情绪不好在多大程度上影响了家人朋友、邻居或集体的正常社交活动？

请打一个钩

根本没有影响	○
很少有影响	○
中度有影响	○
有较大影响	○
有极大影响	○

7. 在过去四个星期里，您有身体上疼痛吗？

请打一个钩

根本没有疼痛	○
有很轻微疼痛	○
有轻微疼痛	○
有中度疼痛	○
有严重疼痛	○
有很严重疼痛	○

8. 在过去四个星期里，身体上的疼痛影响了您的正常工作了吗（包括上班工作和家务活）？

请打一个钩

根本没有影响	○
很少有影响	○
中度有影响	○
有较大影响	○
有极大影响	○

您的感觉

9. 以下这些问题有关过去一个月里您的感觉如何以及您的情况如何。（对每一条问题，请钩出最接近您的感觉的那个答案）

请在每一行打一个钩

在过去一个月里持续的时间	所有的时间	大部分时间	比较多时间	一部分时间	没有此感觉
（1）您觉得生活充实吗？	○	○	○	○	○
（2）您是一个精神紧张的人吗？	○	○	○	○	○
（3）您感到垂头丧气，什么事都不能使你振作起来吗？	○	○	○	○	○
（4）您觉得心情平静吗？	○	○	○	○	○
（5）您精力充沛吗？	○	○	○	○	○
（6）您的情绪低落吗？	○	○	○	○	○
（7）您觉得筋疲力尽吗？	○	○	○	○	○
（8）您是个快乐的人吗？	○	○	○	○	○

（9）您感觉疲劳吗？	○	○	○	○	○

10. 过去的四周内，您的健康或情绪问题是否影响了您的社交活动（比如走亲访友）？

请打一个钩

所有的时间	○
大部分时间	○
比较多时间	○
一部分时间	○
根本没有	○

总的健康情况

11. 请对下面的每一句话，选出最符合您情况的答案

请在每一行打一个钩

	绝对正确	大部分正确	不能肯定	一部分错误	绝对错误
（1）我好像比别人容易生病	○	○	○	○	○
（2）我跟我认识的人一样健康	○	○	○	○	○
（3）我认为我的健康状况在变坏	○	○	○	○	○
（4）我的健康状况非常好	○	○	○	○	○

舌脉情况

项目	具体征象
舌面	□正常 □胖大 □瘦小 □老 □嫩 □淡 □紫 □暗 □红 □瘀斑 □瘀点 □齿痕<br>□其他：________
舌下静脉丛	□迂曲 □怒张 □紫红 □绛紫　□其他：________
舌苔	□　正常 □厚 □薄 □少 □剥 □无 □白 □黄 □腻 □少津　其他：________
脉象	□　正常 □沉 □迟 □数 □细 □弦 □弱 □滑 □涩 □促 □结 □代<br>□　其他：________

医院编号	随机号	受试者姓名拼音缩写	组　别
□□□	□□□□	□□□□	□ A组　□ B组

心电图活动平板运动试验检查记录（前 100 名患者行此检查）

陈旧性心梗病史：□0 无 □1 有							冠造或核素检查诊断依据 □0 无 □1 有					
运动前心率：　次/分							运动试验终止的心率　分/次					
**ST 段下降者填下表** □0 无 □1 有→填下表												
ST 段下降 0.1mV 的时间：____分____秒							下降最大的 ST 段恢复到运动前水平所需时间：___分___秒					
运动全过程中心电图 ST 段下降最大幅度（mV）	I		II		III		aVR		aVL		aVF	
	V1		V2		V3		V4		V5		V6	
ST 段最大下降幅度和（mV）	Σ =											
**ST 段抬高者填下表** □0 无 □1 有→填下表							有陈旧心梗者表明梗塞部位：					
心电图 ST 段开始抬高的时间：____分____秒							心电图 ST 段抬高最大幅度的持续时间：____分____秒					
运动全过程中心电图 ST 段抬高最大幅度（mV）	I		II		III		aVR		aVL		aVF	
	V1		V2		V3		V4		V5		V6	
ST 段最大抬高幅度和（mV）	Σ =											
运动试验级别：□级							代谢当量（MET）：					
运动试验全过程的血压监测	运动试验开始时的血压值　mmHg											
	全运动试验中血压上升的最高值（以收缩为标准）　mmHg											
	血压上升到最高值所需时间　分/秒											
	**血压不升或下降者填写下表** □0 否 □1 是→填下表											
	收缩压上升<20mmHg 者的血压值　mmHg，　此时运动级别 □级											
	收缩压下降到最低时血压值　mmHg						下降到最低值时间___分___秒					
运动试验终止的时间　____分____秒												
运动试验终止的原因							心绞痛时使用硝酸甘油：□有　□无					

心绞痛诱发因素　寒冷 □　劳累 □　情志 □　其它 □

普通心电图检测纪录（无ST-T改变者划/）：

各导联ST段下降幅度(mV)	I		II		III		aVR		aVL		aVF	
	V1		V2		V3		V4		V5		V6	
ST 段下降幅度和（mV）	Σ =											
以波为主导联 T 波倒置幅度（mV）	I		II		III		aVR		aVL		aVF	
	V1		V2		V3		V4		V5		V6	
T 波幅度和（mV）	Σ =											

医院编号	随机号	受试者姓名拼音缩写	组 别
□□□	□□□□	□□□□	□ A 组 □ B 组

终点指标 □ 无 □ 有 发生时间：______年____月____日

主要终点指标	是否发生	次要终点指标	是否发生
心血管死亡	是□ 否□	脑卒中	是□ 否□
非致命性心肌梗死	是□ 否□	因 ACS 再住院	是□ 否□
再次介入治疗	是□ 否□	肺栓塞	是□ 否□
冠脉搭桥术	是□ 否□	外周血管事件	是□ 否□
		全因死亡	是□ 否□
其它		其它	

不良反应记录表 □ 有 □ 无

不良反应	发生时间	终止时间	持续时间	程 度	缓解方式
1				□轻度为 1，中度为 2，重度为 3	□自行缓解为 1，用药缓解为 2，未缓解为 3
2				□轻度为 1，中度为 2，重度为 3	□自行缓解为 1，用药缓解为 2，未缓解为 3
3				□轻度为 1，中度为 2，重度为 3	□自行缓解为 1，用药缓解为 2，未缓解为 3
4				□轻度为 1，中度为 2，重度为 3	□自行缓解为 1，用药缓解为 2，未缓解为 3

依从性评定

随诊方式	按时服药	按量服药	服用其它药物	药瓶回收	继发药物
□ 门诊<br>□ 电话	□ 是<br>□ 否	□ 是<br>□ 否	□ 是<br>□ 否 ____	□ 是<br>□ 否	□ 是<br>□ 否

药物返还： □ 是 □ 否

返还药物数量 □□□盒

药物发放：□ 是 □ 否

发放药物数量 □□□盒

日期_____年_____月_____日

研究者：__________ 填表日期：_____年____月____日

检验报告粘贴单

病例观察记录表（四）（治疗 24 周）

查体： 心率：□□□次/分　　心律：□齐 □不齐______________________________

血压：□□□/□□□mmHg

有无出血点或瘀斑　　□无；　□有　___________________

其它阳性体征　　□无；　□有　___________________

中医症状积分（包括中医兼证）

中医症状	无	轻　度（1 分）	中　度（2 分）	重　度（3 分）
形体肥胖	□#	□体重指数＞25	□□体重指数＞30	□□体重指数＞35
胸闷	□	□胸闷轻微	□明显，有时叹息	□胸闷如窒，叹息不止
固定性刺痛	□	□刺痛轻微，可自行缓解	□刺痛明显，需含药缓解	□刺痛重，持续不缓解
气短	□	□日常活动，感气短	□轻微活动，感气短	□不活动，感气短
乏力	□	□日常活动，感乏力	□轻微活动，感乏力	□不活动，感乏力
口干不欲饮	□	□口干，可忍受	□口干，常需漱口	□ 持续口干，夜间尤甚
口唇紫暗	□	□口唇稍暗	□口唇紫暗	□口唇紫暗发黑
爪甲紫暗	□	□爪甲稍暗	□爪甲紫暗	□爪甲紫暗发黑
肢体沉重	□	□活动时，觉肢体沉重	□轻微活动，感肢体沉重	□不活动，感肢体沉重
四肢困倦	□	□困倦，不影响工作	□困倦，影响工作	□困倦，严重影响工作
口中粘腻	□	□口中粘腻，稍有不爽	□口中粘腻，严重不爽	□口中粘腻，偶有痰涎
其他	□	□	□	□
其他	□	□	□	□
其他	□	□	□	□

#体重指数≤25

证候分级：轻□ 中□ 重□。分级标准：轻：总分≤11 分；中：总分 12-21 分；重：总分≥22 分。

医院编号	随机号	受试者姓名拼音缩写	组　别
□□□	□□□□	□□□□	□ A 组　□ B 组

生活质量自评量表-简明健康调查问卷(SF-36)

下面的问题是询问您对自已健康状况的看法、您的感觉如何、以及您进行日常活动的能力如何，每个问题请选择一个最适合您的答案，并在答案后的“○”中打“√”。

1.总体来讲，您的健康状况是：

	请打一个钩
非常好	○
很好	○
好	○
一般	○
差	○

2.跟一年前相比，您觉得您现在的健康状况是：

	请打一个钩
比一年前好多了	○
比一年前好一些	○
和一年前差不多	○
比一年前差一些	○
比一年前差多了	○

健康和日常活动

3. 以下这些活动都与日常活动有关，您的健康状况是否限制了这些活动？如果有限制，程度如何？

	有很多限制	*有一定限制*	*根本没限制*
（1）重体力活动（跑步、举重、激烈运动）	○	○	○
（2）适度活动（如移桌子、扫地、做操）	○	○	○
（3）手提或搬运日常用品（如买菜、购物）	○	○	○
（4）上几层楼梯	○	○	○
（5）上一层楼梯	○	○	○
（6）弯腰、屈膝、下蹲	○	○	○
（7）步行 1500 米左右的路程	○	○	○
（8）步行 800 米左右的路程	○	○	○
（9）步行约 100 米的路程	○	○	○
（10）自己洗澡、穿衣	○	○	○

4.在过去四个星期里，您的工作和日常活动有没有因为身体的原因而出现以下这些问题？

每个问题都回答有或没

	有	没有
（1）减少了工作或其他活动的时间	○	○
（2）本来想要做的事情减少了一部分	○	○
（3）想要做的工作或活动的种类受到限制	○	○
（4）完成工作或其他活动有困难（比如需要额外的努力）	○	○

5. 在过去四个星期里，您的工作和日常活动有没有因为情绪（如感到消沉或忧虑）的原因而出现以下这些问题？

每个问题都回答有或没

	有	没有
（1）减少了工作或其他活动的时间	○	○
（2）本来想要做的事情只能完成一部分	○	○
（3）做工作或其他活动不如平常仔细	○	○

6. 在过去四个星期里，您的身体健康和情绪不好在多大程度上影响了家人朋友、邻居或集体的正常社交活动？

请打一个钩

根本没有影响	○
很少有影响	○
中度有影响	○
有较大影响	○
有极大影响	○

7. 在过去四个星期里，您有身体上疼痛吗？

请打一个钩

根本没有疼痛	○
有很轻微疼痛	○
有轻微疼痛	○
有中度疼痛	○
有严重疼痛	○
有很严重疼痛	○

8. 在过去四个星期里，身体上的疼痛影响了您的正常工作了吗（包括上班工作和家务活）？

请打一个钩

根本没有影响	○
很少有影响	○
中度有影响	○
有较大影响	○
有极大影响	○

您的感觉

9. 以下这些问题有关过去一个月里您的感觉如何以及您的情况如何。（对每一条问题，请钩出最接近您的感觉的那个答案）

请在每一行打一个钩

在过去一个月里持续的时间	所有的时间	大部分时间	比较多时间	一部分时间	没有此感觉
（1）您觉得生活充实吗？	○	○	○	○	○
（2）您是一个精神紧张的人吗？	○	○	○	○	○
（3）您感到垂头丧气，什么事都不能使你振作起来吗？	○	○	○	○	○
（4）您觉得心情平静吗？	○	○	○	○	○
（5）您精力充沛吗？	○	○	○	○	○
（6）您的情绪低落吗？	○	○	○	○	○
（7）您觉得筋疲力尽吗？	○	○	○	○	○
（8）您是个快乐的人吗？	○	○	○	○	○
（9）您感觉疲劳吗？	○	○	○	○	○

10. 过去的四周内，您的健康或情绪问题是否影响了您的社交活动（比如走亲访友）？

请打一个钩

所有的时间	○
大部分时间	○
比较多时间	○
一部分时间	○
根本没有	○

总的健康情况

11. 请对下面的每一句话，选出最符合您情况的答案

请在每一行打一个钩

	绝对正确	大部分正确	不能肯定	一部分错误	绝对错误
（1）我好像比别人容易生病	○	○	○	○	○
（2）我跟我认识的人一样健康	○	○	○	○	○
（3）我认为我的健康状况在变坏	○	○	○	○	○
（4）我的健康状况非常好	○	○	○	○	○

舌脉情况

项目	具体征象
舌面	□正常 □胖大 □瘦小 □老 □嫩 □淡 □紫 □暗 □红 □瘀斑 □瘀点 □齿痕<br>□其他：________
舌下静脉丛	□迂曲 □怒张 □紫红 □绛紫　□其他：________
舌苔	□　正常 □厚 □薄 □少 □剥 □无 □白 □黄 □腻 □少津　其他：________
脉象	□　正常 □沉 □迟 □数 □细 □弦 □弱 □滑 □涩 □促 □结 □代<br>□　其他：________

理化检查(_____年__月__日)：

检测项目		结 果	指标单位	检测项目	结 果	指标单位
血常规	RBC		$\times 10^{12}$/L	Hb		g/L
	WBC		$\times 10^{9}$/L	PLT		$\times 10^{9}$/L
	PDW		fL	N		%
	L		%			
尿常规	白细胞		个/HP	红细胞		个/HP
	蛋白质		g/L	葡萄糖		mmol/L
便常规	白细胞		个/HP	红细胞		个/HP
	便潜血		定性			
生化指标	ALT		U/L	AST		U/L
	BUN		mmol/L	Cr		μmol/L
	GRF		%	TC		mmol/L
	TG		mmol/L	LDL-C		mmol/L
	GLU		mmol/L	HDL-C		mmol/L
凝血四项	PT		s	APTT		s
	Fbg		g/L	TT		s
心功能	BNP		ng/l	Pro-BNP		ng/L
炎症	CRP		mg/l	高敏 CRP		mg/l

医院编号	随机号	受试者姓名拼音缩写	组　别
□□□	□□□□	□□□□	□ A组　□ B组

心电图活动平板运动试验检查记录（前 100 名患者行此检查）

陈旧性心梗病史：□0 无 □1 有							冠造或核素检查诊断依据　□0 无 □1 有					
运动前心率：　次/分							运动试验终止的心率　分/次					
**ST 段下降者填下表**　□0 无 □1 有→填下表												
ST 段下降 0.1mV 的时间：____分____秒							下降最大的 ST 段恢复到运动前水平所需时间：___分___秒					
运动全过程中心电图 ST 段下降最大幅度（mV）	I		II		III		aVR		aVL		aVF	
	V1		V2		V3		V4		V5		V6	
ST 段最大下降幅度和（mV）	Σ =											
**ST 段抬高者填下表**　□0 无 □1 有→填下表							有陈旧心梗者表明梗塞部位：					
心电图 ST 段开始抬高的时间：____分____秒							心电图 ST 段抬高最大幅度的持续时间：____分____秒					
运动全过程中心电图 ST 段抬高最大幅度（mV）	I		II		III		aVR		aVL		aVF	
	V1		V2		V3		V4		V5		V6	
ST 段最大抬高幅度和（mV）	Σ =											
运动试验级别：　□级							代谢当量（MET）：					
运动试验全过程的血压监测	运动试验开始时的血压值　mmHg											
	全运动试验中血压上升的最高值（以收缩为标准）　mmHg											
	血压上升到最高值所需时间　分/秒											
	**血压不升或下降者填写下表**　□0 否　□1 是→填下表											
	收缩压上升<20mmHg 者的血压值　mmHg，　此时运动级别　□级											
	收缩压下降到最低时血压值　mmHg						下降到最低值时间___ 分___秒					
运动试验终止的时间　____ 分____秒												
运动试验终止的原因							心绞痛时使用硝酸甘油：　□有　□无					

心绞痛诱发因素　　寒冷 □　　劳累 □　　情志 □　　其它 □

普通心电图检测纪录（无ST-T改变者划/）：

各导联ST段下降幅度(mV)	I		II		III		aVR		aVL		aVF	
	V1		V2		V3		V4		V5		V6	
ST 段下降幅度和（mV）	Σ =											
以波为主导联 T 波倒置幅度（mV）	I		II		III		aVR		aVL		aVF	
	V1		V2		V3		V4		V5		V6	
T 波幅度和（mV）	Σ =											

医院编号	随机号	受试者姓名拼音缩写	组　别
□□□	□□□□	□□□□	□ A组　□ B组

心电图(_____年____月____日)

心率：□□□次/分；基础心律：□窦性　□房颤　□室早　其他________________

□心律失常：□室早　□室上早　□室速　□室上速

□房室传导阻滞：□Ⅰ度 □Ⅱ度Ⅰ型 □Ⅱ度Ⅱ型　□Ⅲ度

□LBBB　□RBBB　□LAHB　□LPHB

□其它________________

□ST 改变：

□下斜性下移：□Ⅰ□Ⅱ□Ⅲ□aVR□aVL□aVF□V1□V2□V3□V4□V5□V6，最大下移_____mV。

□水平性下移：□Ⅰ□Ⅱ□Ⅲ□aVR□aVL□aVF□V1□V2□V3□V4□V5□V6，最大下移_____mV。

□抬高：□Ⅰ□Ⅱ□Ⅲ□aVR□aVL□aVF□V1□V2□V3□V4□V5□V6 最大抬高_____mV。

□其它描述：____

超声心动图(_____年____月____日)：

□心内结构及血流未见异常

□异常：

□室壁运动异常

□室壁节段性或反向运动，部位：□前间壁 □前壁 □广泛前壁 □下壁

□侧壁　□高侧壁 □正后壁

□室壁运动消失，部位：□前间壁 □前壁 □广泛前壁 □下壁

□侧壁　□高侧壁 □正后壁

□室壁运动减弱，部位：□前间壁 □前壁 □广泛前壁 □下壁

□侧壁　□高侧壁 □正后壁

□瓣膜关闭不全或返流

□二尖瓣　□三尖瓣　□主动脉瓣　□肺动脉瓣

□其他描述：____

检测值：左室舒张末期内径：□□□mm；EF：□□%；

医院编号	随机号	受试者姓名拼音缩写	组　别
□□□	□□□□	□□□□	□ A组　□ B组

终点指标　　□ 无　　□ 有　　发生时间：______年____月____日

主要终点指标	是否发生	次要终点指标	是否发生
心血管死亡	是□　否□	脑卒中	是□　否□
非致命性心肌梗死	是□　否□	因 ACS 再住院	是□　否□
再次介入治疗	是□　否□	肺栓塞	是□　否□
冠脉搭桥术	是□　否□	外周血管事件	是□　否□
		全因死亡	是□　否□
其它		其它	

不良反应记录表　　□ 有　　□ 无

不良反应	发生时间	终止时间	持续时间	程　度	缓解方式
1				□轻度为 1，中度为 2，重度为 3	□自行缓解为 1，用药缓解为 2，未缓解为 3
2				□轻度为 1，中度为 2，重度为 3	□自行缓解为 1，用药缓解为 2，未缓解为 3
3				□轻度为 1，中度为 2，重度为 3	□自行缓解为 1，用药缓解为 2，未缓解为 3
4				□轻度为 1，中度为 2，重度为 3	□自行缓解为 1，用药缓解为 2，未缓解为 3

依从性评定

随诊方式	按时服药	按量服药	服用其它药物	药瓶回收	继发药物
□ 门诊<br>□ 电话	□ 是<br>□ 否	□ 是<br>□ 否	□ 是<br>□ 否 ____	□ 是<br>□ 否	□ 是<br>□ 否

药物返还：□ 是　□ 否

返还药物数量　□□□盒

药物发放：□ 是　□ 否

发放药物数量　□□□盒

日期______年______月______日

研究者：____________　　填表日期：_____年____月____日

检验报告粘贴单

医院编号	随机号	受试者姓名拼音缩写	组　别
□□□	□□□□	□□□□	□ A组　□ B组

病例观察记录表（五）（随访 12 周）

查体： 心率：□□□次/分　心律：□齐 □不齐______________________________

血压：□□□/□□□mmHg

有无出血点或瘀斑　□无；　□有　___________________

其它阳性体征　□无；　□有　___________________

中医症状积分（包括中医兼证）

中医症状	无	轻　度（1分）	中　度（2分）	重　度（3分）
形体肥胖	□#	□体重指数＞25	□□体重指数＞30	□□体重指数＞35
胸闷	□	□胸闷轻微	□明显，有时叹息	□胸闷如窒，叹息不止
固定性刺痛	□	□刺痛轻微，可自行缓解	□刺痛明显，需含药缓解	□刺痛重，持续不缓解
气短	□	□日常活动，感气短	□轻微活动，感气短	□不活动，感气短
乏力	□	□日常活动，感乏力	□轻微活动，感乏力	□不活动，感乏力
口干不欲饮	□	□口干，可忍受	□口干，常需漱口	□ 持续口干，夜间尤甚
口唇紫暗	□	□口唇稍暗	□口唇紫暗	□口唇紫暗发黑
爪甲紫暗	□	□爪甲稍暗	□爪甲紫暗	□爪甲紫暗发黑
肢体沉重	□	□活动时，觉肢体沉重	□轻微活动，感肢体沉重	□不活动，感肢体沉重
四肢困倦	□	□困倦，不影响工作	□困倦，影响工作	□困倦，严重影响工作
口中粘腻	□	□口中粘腻，稍有不爽	□口中粘腻，严重不爽	□口中粘腻，偶有痰涎
其他	□	□	□	□
其他	□	□	□	□
其他	□	□	□	□

#体重指数≤25

证候分级：轻□ 中□ 重□。分级标准：轻：总分≤11 分；中：总分 12-21 分；重：总分≥22 分。

生活质量自评量表-简明健康调查问卷(SF-36)

下面的问题是询问您对自已健康状况的看法、您的感觉如何、以及您进行日常活动的能力如何，每个问题请选择一个最适合您的答案，并在答案后的“○”中打“√”。

1.总体来讲，您的健康状况是：

	请打一个钩
非常好	○
很好	○
好	○
一般	○
差	○

2.跟一年前相比，您觉得您现在的健康状况是：

	请打一个钩
比一年前好多了	○
比一年前好一些	○
和一年前差不多	○
比一年前差一些	○
比一年前差多了	○

健康和日常活动

3. 以下这些活动都与日常活动有关，您的健康状况是否限制了这些活动？如果有限制，程度如何？

	有很多限制	*有一定限制*	*根本没限制*
（1）重体力活动（跑步、举重、激烈运动）	○	○	○
（2）适度活动（如移桌子、扫地、做操）	○	○	○
（3）手提或搬运日常用品（如买菜、购物）	○	○	○
（4）上几层楼梯	○	○	○
（5）上一层楼梯	○	○	○
（6）弯腰、屈膝、下蹲	○	○	○
（7）步行 1500 米左右的路程	○	○	○
（8）步行 800 米左右的路程	○	○	○
（9）步行约 100 米的路程	○	○	○
（10）自己洗澡、穿衣	○	○	○

4.在过去四个星期里，您的工作和日常活动有没有因为身体的原因而出现以下这些问题？

每个问题都回答有或没

	有	没有
（1）减少了工作或其他活动的时间	○	○
（2）本来想要做的事情减少了一部分	○	○
（3）想要做的工作或活动的种类受到限制	○	○
（4）完成工作或其他活动有困难（比如需要额外的努力）	○	○

5. 在过去四个星期里，您的工作和日常活动有没有因为情绪（如感到消沉或忧虑）的原因而出现以下这些问题？

每个问题都回答有或没

	有	没有
（1）减少了工作或其他活动的时间	○	○
（2）本来想要做的事情只能完成一部分	○	○
（3）做工作或其他活动不如平常仔细	○	○

6. 在过去四个星期里，您的身体健康和情绪不好在多大程度上影响了家人朋友、邻居或集体的正常社交活动？

请打一个钩

根本没有影响	○
很少有影响	○
中度有影响	○
有较大影响	○
有极大影响	○

7. 在过去四个星期里，您有身体上疼痛吗？

请打一个钩

根本没有疼痛	○
有很轻微疼痛	○
有轻微疼痛	○
有中度疼痛	○
有严重疼痛	○
有很严重疼痛	○

8. 在过去四个星期里，身体上的疼痛影响了您的正常工作了吗（包括上班工作和家务活）？

请打一个钩

根本没有影响	○
很少有影响	○
中度有影响	○
有较大影响	○
有极大影响	○

您的感觉

9. 以下这些问题有关过去一个月里您的感觉如何以及您的情况如何。（对每一条问题，请钩出最接近您的感觉的那个答案）

请在每一行打一个钩

在过去一个月里持续的时间	所有的时间	大部分时间	比较多时间	一部分时间	没有此感觉
（1）您觉得生活充实吗？	○	○	○	○	○
（2）您是一个精神紧张的人吗？	○	○	○	○	○
（3）您感到垂头丧气，什么事都不能使你振作起来吗？	○	○	○	○	○
（4）您觉得心情平静吗？	○	○	○	○	○
（5）您精力充沛吗？	○	○	○	○	○
（6）您的情绪低落吗？	○	○	○	○	○
（7）您觉得筋疲力尽吗？	○	○	○	○	○
（8）您是个快乐的人吗？	○	○	○	○	○
（9）您感觉疲劳吗？	○	○	○	○	○

10. 过去的四周内，您的健康或情绪问题是否影响了您的社交活动（比如走亲访友）？

请打一个钩

所有的时间	○
大部分时间	○
比较多时间	○
一部分时间	○
根本没有	○

总的健康情况

11.请对下面的每一句话，选出最符合您情况的答案

请在每一行打一个钩

	绝对<br>正确	大部分<br>正确	不能<br>肯定	一部分<br>错误	绝对<br>错误
（1）我好像比别人容易生病	○	○	○	○	○
（2）我跟我认识的人一样健康	○	○	○	○	○
（3）我认为我的健康状况在变坏	○	○	○	○	○
（4）我的健康状况非常好	○	○	○	○	○

舌脉情况

项目	具体征象
舌面	□正常 □胖大 □瘦小 □老 □嫩 □淡 □紫 □暗 □红 □瘀斑 □瘀点 □齿痕<br>□其他：________
舌下静脉丛	□迂曲 □怒张 □紫红 □绛紫　□其他：________
舌苔	□　正常 □厚 □薄 □少 □剥 □无 □白 □黄 □腻 □少津　其他：________
脉象	□　正常 □沉 □迟 □数 □细 □弦 □弱 □滑 □涩 □促 □结 □代<br>□　其他：________

终点指标　　□ 无　　□ 有　　发生时间：______年____月____日

主要终点指标	是否发生	次要终点指标	是否发生
心血管死亡	是□　　否□	脑卒中	是□　　否□
非致命性心肌梗死	是□　　否□	因 ACS 再住院	是□　　否□
再次介入治疗	是□　　否□	肺栓塞	是□　　否□
冠脉搭桥术	是□　　否□	外周血管事件	是□　　否□
		全因死亡	是□　　否□
其它		其它	

研究者：____________　　　　填表日期：_____年____月____日

用药前检验报告粘贴单

病例观察记录表（六）（随访 24 周）

查体： 心率：□□□次/分 心律：□齐 □不齐______________________________

血压：□□□/□□□mmHg

有无出血点或瘀斑 □无； □有 ___________________

其它阳性体征 □无； □有 ___________________

中医症状积分（包括中医兼证）

中医症状	无	轻 度（1 分）	中 度（2 分）	重 度（3 分）
形体肥胖	□#	□体重指数＞25	□□体重指数＞30	□□体重指数＞35
胸闷	□	□胸闷轻微	□明显，有时叹息	□胸闷如窒，叹息不止
固定性刺痛	□	□刺痛轻微，可自行缓解	□刺痛明显，需含药缓解	□刺痛重，持续不缓解
气短	□	□日常活动，感气短	□轻微活动，感气短	□不活动，感气短
乏力	□	□日常活动，感乏力	□轻微活动，感乏力	□不活动，感乏力
口干不欲饮	□	□口干，可忍受	□口干，常需漱口	□ 持续口干，夜间尤甚
口唇紫暗	□	□口唇稍暗	□口唇紫暗	□口唇紫暗发黑
爪甲紫暗	□	□爪甲稍暗	□爪甲紫暗	□爪甲紫暗发黑
肢体沉重	□	□活动时，觉肢体沉重	□轻微活动，感肢体沉重	□不活动，感肢体沉重
四肢困倦	□	□困倦，不影响工作	□困倦，影响工作	□困倦，严重影响工作
口中粘腻	□	□口中粘腻，稍有不爽	□口中粘腻，严重不爽	□口中粘腻，偶有痰涎
其他	□	□	□	□
其他	□	□	□	□
其他	□	□	□	□

#体重指数≤25

证候分级：轻□ 中□ 重□。分级标准：轻：总分≤11 分；中：总分 12-21 分；重：总分≥22 分。

生活质量自评量表-简明健康调查问卷(SF-36)

下面的问题是询问您对自己健康状况的看法、您的感觉如何、以及您进行日常活动的能力如何，每个问题请选择一个最适合您的答案，并在答案后的“○”中打“√”。

1.总体来讲，您的健康状况是：

	请打一个钩
非常好	○
很好	○
好	○
一般	○
差	○

2.跟一年前相比，您觉得您现在的健康状况是：

	请打一个钩
比一年前好多了	○
比一年前好一些	○
和一年前差不多	○
比一年前差一些	○
比一年前差多了	○

健康和日常活动

3. 以下这些活动都与日常活动有关，您的健康状况是否限制了这些活动？如果有限制，程度如何？

	有很多限制	*有一定限制*	*根本没限制*
（1）重体力活动（跑步、举重、激烈运动）	○	○	○
（2）适度活动（如移桌子、扫地、做操）	○	○	○
（3）手提或搬运日常用品（如买菜、购物）	○	○	○
（4）上几层楼梯	○	○	○
（5）上一层楼梯	○	○	○
（6）弯腰、屈膝、下蹲	○	○	○
（7）步行 1500 米左右的路程	○	○	○
（8）步行 800 米左右的路程	○	○	○
（9）步行约 100 米的路程	○	○	○
（10）自己洗澡、穿衣	○	○	○

4.在过去四个星期里，您的工作和日常活动有没有因为身体的原因而出现以下这些问题？

每个问题都回答有或没

	有	没有
（1）减少了工作或其他活动的时间	○	○
（2）本来想要做的事情减少了一部分	○	○
（3）想要做的工作或活动的种类受到限制	○	○
（4）完成工作或其他活动有困难（比如需要额外的努力）	○	○

5. 在过去四个星期里，您的工作和日常活动有没有因为情绪（如感到消沉或忧虑）的原因而出现以下这些问题？

每个问题都回答有或没

	有	没有
（1）减少了工作或其他活动的时间	○	○
（2）本来想要做的事情只能完成一部分	○	○
（3）做工作或其他活动不如平常仔细	○	○

6. 在过去四个星期里，您的身体健康和情绪不好在多大程度上影响了家人朋友、邻居或集体的正常社交活动？

请打一个钩

根本没有影响	○
很少有影响	○
中度有影响	○
有较大影响	○
有极大影响	○

7. 在过去四个星期里，您有身体上疼痛吗？

请打一个钩

根本没有疼痛	○
有很轻微疼痛	○
有轻微疼痛	○
有中度疼痛	○
有严重疼痛	○
有很严重疼痛	○

8. 在过去四个星期里，身体上的疼痛影响了您的正常工作了吗（包括上班工作和家务活）？

请打一个钩

根本没有影响	○
很少有影响	○
中度有影响	○
有较大影响	○
有极大影响	○

您的感觉

9. 以下这些问题有关过去一个月里您的感觉如何以及您的情况如何。（对每一条问题，请钩出最接近您的感觉的那个答案）

请在每一行打一个钩

在过去一个月里持续的时间	所有的时间	大部分时间	比较多时间	一部分时间	没有此感觉
（1）您觉得生活充实吗？	○	○	○	○	○
（2）您是一个精神紧张的人吗？	○	○	○	○	○
（3）您感到垂头丧气，什么事都不能使你振作起来吗？	○	○	○	○	○
（4）您觉得心情平静吗？	○	○	○	○	○
（5）您精力充沛吗？	○	○	○	○	○
（6）您的情绪低落吗？	○	○	○	○	○
（7）您觉得筋疲力尽吗？	○	○	○	○	○
（8）您是个快乐的人吗？	○	○	○	○	○
（9）您感觉疲劳吗？	○	○	○	○	○

10. 过去的四周内，您的健康或情绪问题是否影响了您的社交活动（比如走亲访友）？

请打一个钩

所有的时间	○
大部分时间	○
比较多时间	○
一部分时间	○
根本没有	○

总的健康情况

11. 请对下面的每一句话，选出最符合您情况的答案

请在每一行打一个钩

	绝对正确	大部分正确	不能肯定	一部分错误	绝对错误
（1）我好像比别人容易生病	○	○	○	○	○
（2）我跟我认识的人一样健康	○	○	○	○	○
（3）我认为我的健康状况在变坏	○	○	○	○	○
（4）我的健康状况非常好	○	○	○	○	○

舌脉情况

项目	具体征象
舌面	□正常 □胖大 □瘦小 □老 □嫩 □淡 □紫 □暗 □红 □瘀斑 □瘀点 □齿痕<br>□其他：________________
舌下静脉丛	□迂曲 □怒张 □紫红 □绛紫　□其他：________________
舌苔	□　正常 □厚 □薄 □少 □剥 □无 □白 □黄 □腻 □少津　其他：______________
脉象	□　正常 □沉 □迟 □数 □细 □弦 □弱 □滑 □涩 □促 □结 □代<br>□　其他：________________

医院编号	随机号	受试者姓名拼音缩写	组　别
□□□	□□□□	□□□□	□ A组　□ B组

理化检查(_____年__月__日)：

检测项目		结　果	指标单位	检测项目	结　果	指标单位
血常规	RBC		×10^{12}/L	Hb		g/L
	WBC		×10^{9}/L	PLT		×10^{9}/L
	PDW		fL	N		%
	L		%			
尿常规	白细胞		个/HP	红细胞		个/HP
	蛋白质		g/L	葡萄糖		mmol/L
便常规	白细胞		个/HP	红细胞		个/HP
	便潜血		定性			
生化指标	ALT		U/L	AST		U/L
	BUN		mmol/L	Cr		μmol/L
	GRF		%	TC		mmol/L
	TG		mmol/L	LDL-C		mmol/L
	GLU		mmol/L	HDL-C		mmol/L
凝血四项	PT		s	APTT		s
	Fbg		s	TT		s
心功能	BNP		ng/l	Pro-BNP		ng/l
炎症	CRP		mg/l	高敏 CRP		mg/l

心电图(_____年____月____日)

心率：□□□次/分；基础心律：□窦性 □房颤 □室早 其他________________

□心律失常：□室早 □室上早 □室速 □室上速

□房室传导阻滞：□Ⅰ度 □Ⅱ度Ⅰ型 □Ⅱ度Ⅱ型 □Ⅲ度

□LBBB □RBBB □LAHB □LPHB

□其它________________

□ST 改变：

□下斜性下移：□Ⅰ□Ⅱ□Ⅲ□aVR□aVL□aVF□V1□V2□V3□V4□V5□V6，最大下移_____mV。

□水平性下移：□Ⅰ□Ⅱ□Ⅲ□aVR□aVL□aVF□V1□V2□V3□V4□V5□V6，最大下移_____mV。

□抬高：□Ⅰ□Ⅱ□Ⅲ□aVR□aVL□aVF□V1□V2□V3□V4□V5□V6 最大抬高_____mV。

□其它描述：____

超声心动图(_____年____月____日)：

□心内结构及血流未见异常

□异常：

□室壁运动异常

□室壁节段性或反向运动，部位：□前间壁 □前壁 □广泛前壁 □下壁
□侧壁 □高侧壁 □正后壁

□室壁运动消失，部位：□前间壁 □前壁 □广泛前壁 □下壁
□侧壁 □高侧壁 □正后壁

□室壁运动减弱，部位：□前间壁 □前壁 □广泛前壁 □下壁
□侧壁 □高侧壁 □正后壁

□瓣膜关闭不全或返流

□二尖瓣 □三尖瓣 □主动脉瓣 □肺动脉瓣

□其他描述：____

检测值：左室舒张末期内径：□□□mm；EF：□□%；

医院编号	随机号	受试者姓名拼音缩写	组　别
□□□	□□□□	□□□□	□ A组　□ B组

终点指标　　□ 无　　□ 有　　发生时间：______年____月____日

主要终点指标	是否发生	次要终点指标	是否发生
心血管死亡	是□　否□	脑卒中	是□　否□
非致命性心肌梗死	是□　否□	因 ACS 再住院	是□　否□
再次介入治疗	是□　否□	肺栓塞	是□　否□
冠脉搭桥术	是□　否□	外周血管事件	是□　否□
		全因死亡	是□　否□
其它		其它	

研究者：______________　　　　填表日期：______年____月____日

检验报告粘贴单

医院编号	随机号	受试者姓名拼音缩写	组　别
□□□	□□□□	□□□□	□ A 组　□ B 组

临床试验完成情况总结

终点指标　　□ 无　　□ 有　　发生时间：______年____月____日

主要终点指标	是否发生	次要终点指标	是否发生
心血管死亡	是□　否□	脑卒中	是□　否□
非致命性心肌梗死	是□　否□	因 ACS 再住院	是□　否□
再次介入治疗	是□　否□	肺栓塞	是□　否□
冠脉搭桥术	是□　否□	外周血管事件	是□　否□
		全因死亡	是□　否□
其它		其它	

安全性评价

□1 级：安全，无任何不良反应；安全性指标检查无异常。

□2 级：比较安全，有轻度不良反应，不需做任何处理可继续给药，安全性指标检查无异常。

□3 级：有安全性问题，有中等程度的不良反应，或安全性指标检查有轻度异常，做处理后可继续给药。

□4 级：因严重不良反应中止试验；或安全性指标检查明显异常。

受试者的随机号是否被揭盲：□ 是；□ 否

如否，请填写以下项目：

受试者是否中止临床试验？　□ 是；□ 否

中止临床试验的日期：

中止临床试验的原因：□

1. 不良事件(已填写不良事件表)
2. 缺乏疗效
3. 违背试验方案
4. 失访
5. 受试者不合作
6. 出现其他并发症
7. 其它(请详述) ________________________________

中止临床试验时的治疗评价：

□ 有效(无再狭窄)；□ 无效(有再狭窄) ；□ 不确定

研究者：____________　　　填表日期：_____年____月____日
