# Supplementary material for: Effect of Xinyue capsules on patients with coronary heart disease after percutaneous coronary intervention: study protocol for a randomized controlled trial
Source: Trials. 2016 Aug 18;17:412. doi: 10.1186/s13063-016-1531-x (PMC4991005; doi:10.1186/s13063-016-1531-x)
Supplement: Additional file 3: — Investigators brochure R2. (PDF 943 kb) [file 13063_2016_1531_MOESM3_ESM.pdf]

吉林省科委创新药物成果转化项目

心悦胶囊治疗介入后冠心病的随机、双盲、安慰剂对照临床试验

研究者手册

课题研究单位：中国中医科学院西苑医院
吉林益盛药业股份有限公司

二零一三年七月

目录

第一部分 立题背景及目的 1

一、立题背景 1

二、试验目的 2

第二部分 研究内容 3

一、相关标准 3

1.中医诊断标准 3

2.中医辨证标准 3

3.西医诊断标准 3

4.纳入标准 3

5.排除标准 4

6.剔除、脱落标准 5

7.试验中止标准 6

8.试验终止标准 6

二、技术路线 7

三、实施流程 8

1. 筛选受试者 8

2. 签署知情同意书 8

3. 药物用法 8

4.药物发放 8

5.药物回收 8

6.合并用药的规定 9
7. 试验指标观测流程图 10
8. 终点指标 11
9. 安全性指标 11
10. 运动耐量指标 11
11.依从性观察...11
12.不良事件与不良反应 12
13.数据录入 13

第一部分 立题背景及目的

一、立题背景

尽管冠心病（coronary heart disease, CHD）尤其是急性冠脉综合征（包括不稳定性心绞痛、非 ST 段抬高型心肌梗死和 ST 段抬高型心肌梗死）药物干预和血运重建治疗获得显著进展，但其仍是威胁人类健康的高发病率和高死亡率疾病[1,2]。2010 年一项由中美合作完成的基于目前心血管病危险因素在中国流行状况的研究估测，从 2010 年到 2030 年，中国心血管病患者将增加 2130 万，心血管病死亡人数将增加 770 万[3]。如何进一步提高 CHD 二级预防疗效，降低心血管事件发生率，是目前亟待解决的关键问题[4]。中医药理应在此方面发挥应有的作用。

心悦胶囊是吉林省集安益盛药业股份有限公司研发生产、具有自主知识产权、2005 年 SFDA 批准上市的治疗 CHD 心绞痛的国家中药新药。以往西洋参入药部位主要为其根部，而心悦胶囊的主要成分则是从西洋参茎叶中提取的西洋参茎叶总皂苷。前期研究证明，西洋参茎叶皂苷含量不低于根部，优化节省了药物资源。心悦胶囊具有有效成分易检测、代谢途径易研究、药效机制较明确的优势。心悦胶囊上市后，吉林省集安益盛药业股份有限公司联合国内外医疗科研机构致力于该药防治 CHD 的一系列实验和临床研究：实验研究证明，心悦胶囊能作用于冠心病发病的多个病理环节，具有抗心肌细胞损伤[5]、保护心脏功能和降低心肌耗氧[6,7]、改善心肌组织血流灌注[8]、改善急性心肌梗死后心室重构[9]、抗缺血心肌细胞凋亡[10,11]、调节糖和脂质代谢[12]、改善胰岛素抵抗[13,14]等作用；II、III 期临床研究证实，心悦胶囊可明显改善 CHD 患者临床心绞痛症状和心电图缺血改变，调节脂质代谢，并具有良好的临床安全性和耐受性[15]。国家“十五”科技攻关项目研究发现，心悦胶囊和活血化瘀中药联合西医常规治疗心肌梗死血运重建后患者，可明显改善患者心脏功能和室壁运动，减少心肌缺血，改善心肌组织灌注和生存质量[16]。国家“十一五”科技支撑计划项目进行设计 13 个中心，800 多例患者的随机对照的临床研究，证实心悦胶囊与活血化瘀中药联合西医常规干预能减少介入治疗术后 CHD 患者 1 年的主要终点事件（心血管死亡、非致命性心

肌梗死、再次血运重建术包括 PCI 和冠脉搭桥）和次要终点事件（脑卒中、因 ACS 再住院、心功能不全和其它血栓并发症）的发生，改善患者生存质量，且无明显不良反应；和奥地利维也纳医科大学合作进行药物化学和药代动力学研究表明，不同批次的心悦胶囊和同一批次不同时间点的心悦胶囊指纹图谱一致，重金属和农残含量低于欧盟标准，且明确了心悦胶囊的主要成分和其代谢途径。

总之，心悦胶囊在基础和临床方面具备了坚实的研究基础，但其对介入治疗后冠心病终点事件和心功能等方面的研究，还未见报道。本研究拟入选介入治疗后冠心病患者，观察心悦胶囊治疗 1 年后终点事件的发生率、心功能的改善情况及临床的安全性，为心悦胶囊联合西医常规干预介入治疗后冠心病提供高水平的证据。

二、 试验目的

- 通过多中心、随机、双盲、安慰剂对照临床试验，进一步明确心悦胶囊治疗介入治疗后冠心病的疗效；
- 通过多中心、大样本观察心悦胶囊临床应用的安全性。

第二部分 研究内容

一、相关标准

1.中医诊断标准

中医病证诊断参照 1997 年国家中医药管理局制定的《中医病证诊断疗效标准》中有关胸痹心痛（冠心病心肌梗死）的诊断标准。见附录 1。

2.中医辨证标准

根据 1993 年《中药新药治疗胸痹（冠心病心绞痛）的临床研究指导原则》中“气阴两虚证”: 胸闷隐痛，时作时止，心悸气短，倦怠懒言，面色少华，头晕目眩，遇劳则甚，舌偏红，脉细弱，或结代。

3.西医诊断标准

参考国际心脏病学会和协会及世界卫生组织临床命名标准化联合专题组报告《缺血性心脏病的命名及诊断标准》、中华医学会心血管病学分会、中华心血管病杂志编辑委员会 2007 年公布的《不稳定型心绞痛和非 ST 段抬高型心肌梗死诊断与治疗指南》和欧洲心脏病学会 2012 年发布的《欧洲心脏病学会 ST 段抬高型心肌梗死管理指南》。

4.纳入标准

（1）符合冠心病诊断，并首次成功行血管介入治疗者；

（2）介入治疗后 1-3 个月病情稳定者：①无进展性胸痛；②肌钙蛋白正常或轻度升高，但低于高值上线 99 百分数；③心绞痛I-II级（CCS 分级）

（3）中医辨证为气阴两虚证的患者；

（4）年龄≤75 岁；

（5）心功能I-II级(NYHA 心功能分级)；

（6）受试者知情，自愿签署知情同意书。

5.排除标准

（1）肾功能不全，男性血清肌酐>2.5 mg/dl（>220umo/l）女性>2.0 mg/dl（>175umo/l）;

（2）患有明显的肝脏疾患或 ALT、AST 高于正常上限 2 倍；

（3）收缩压>160mmHg 或舒张压>100mmHg（测量血压前患者至少需要静坐 5 分钟）;

（4）随机血糖≥13.7mmol/L 的糖尿病患者或糖化血红蛋白≥9.5%；

（5）妊娠或准备妊娠妇女，哺乳期妇女及对研究药物已知成分过敏者；

（6）合并急性脑血管疾病患者；

（7）恶性肿瘤或患者预期寿命少于 3 年；

（8）严重的造血系统疾病患者；

（9）严重的精神病患者；

（10）近三个月内参加过或正在参加其它临床试验者。

6.剔除、脱落标准

6.1 受试者的退出（脱落）

6.1.1 脱落病例判定

所有填写了知情同意书并筛选合格进入随机化试验的受试者，无论何时何因退出，只要没有完成方案所规定的观察周期的受试者，均作为脱落病例。

6.1.2 脱落病例处理

当受试者脱落后，研究者应采取家访、电话、信件等方式随访，尽可能与受试者联系，询问理由，记录最后一次服药时间，完成所能完成的评估项目。因过敏反应、不良反应、治疗无效而退出试验病例，研究者应根据受试者实际情况，采取相应的治疗措施，以保障受试者的权益。脱落病例均应妥善保存有关试验资料，即作留档，也是进行全分析集统计所需。

6.2 受试者剔除

（1）"违反合法性"，即指病例选择违反了纳入/排除病例标准，本不应当进行随机化入组。

（2）受试者不配合随机化入组，或随机化入组后未服用任何试验药物，或用药极少（＜10%）。

（3）使用了禁止合用的其它治疗或药物等，影响有效性和安全性判定者。

（4）在随机化入组之后没有任何数据。

必须在盲态审核时由主要研究者、数据管理员、统计分析专家和申办者共同讨论后，判断该病例是否剔除。

7.试验中止标准

试验中止是指临床试验尚未按方案结束，中途停止全部试验。试验中止的目的主要是为了保护受试者权益，保证试验质量，避免不必要的经济损失。

（1）试验中发生严重安全性问题，应及时中止试验。

（2）试验中发现药物不具有临床价值，应中止试验，避免延误受试者的有效治疗，同时避免不必要的经济损失。

（3）在试验中发现临床试验方案有重大失误，难以评价药物效应；或者一项设计较好的方案，在实施中发生了严重偏差，再继续下去，难以评价药物效应。

（4）申办者因经费、管理等原因要求中止。

（5）国家药品监督管理局因某种原因勒令中止试验。本研究为全国多中心、拟纳入 3000 例患者的前瞻性队列研究。所有参加单位观察的病例数根据其 PCI 例数/年进行适当分配。

8.试验终止标准

（1）研究者判定继续进行研究对您有害；

（2）试验者未按照医生的指导用药；

（3）研究过程中发生了严重不良反应；

（4）临床研究被取消。

二.技术路线

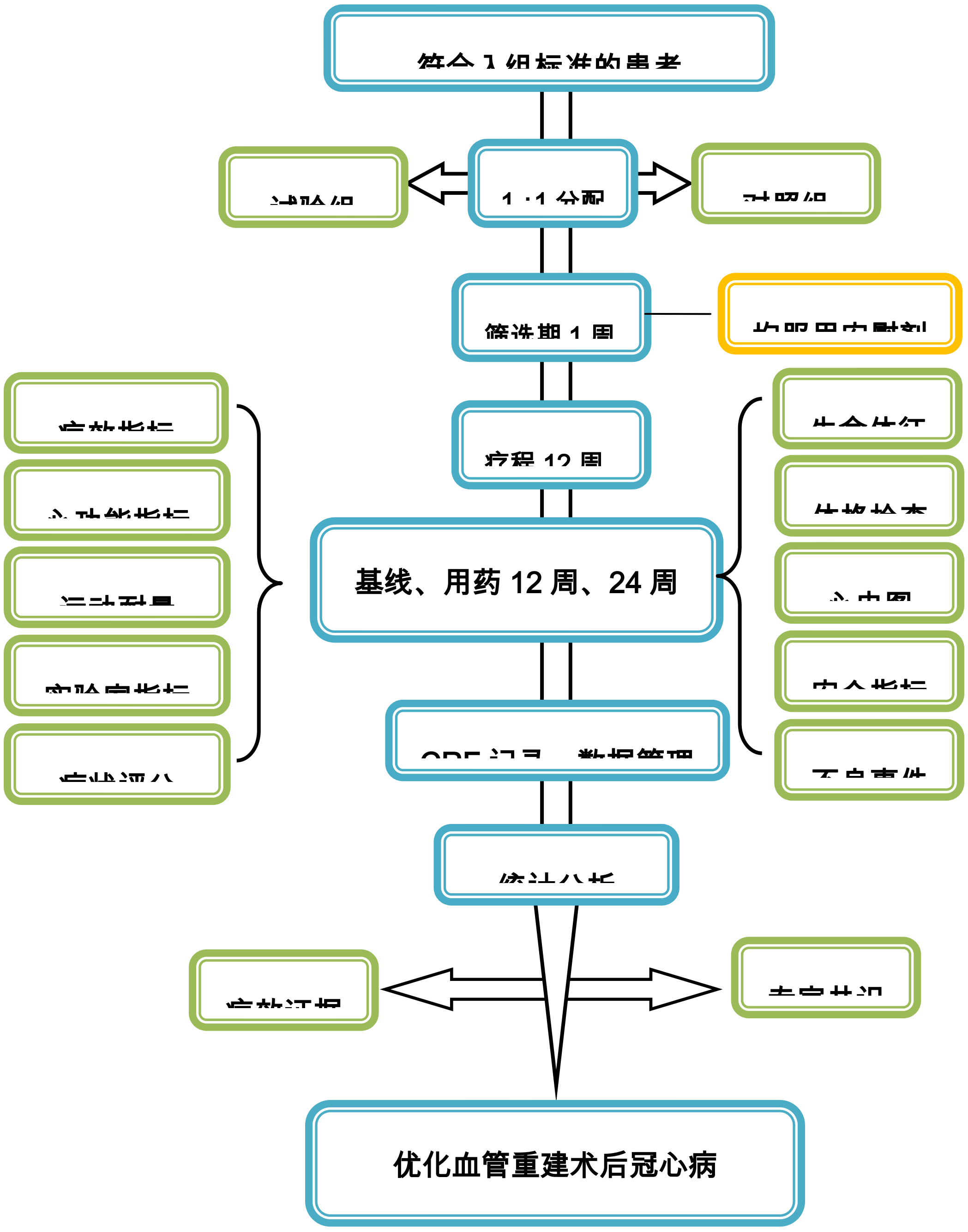

三、实施流程

1. 筛选受试者

根据西医诊断标准、中医诊断标准、纳入标准和排除标准筛选受试者。

2. 签署知情同意书

3. 药物用法

- *筛选期*

每日给予安慰剂，一次 2 粒，一日 3 次，温水冲服，连续 1 周。

- *药物治疗*

试验组每日给予心悦胶囊 2 粒，一日 3 次，温水冲服，连续服用 24 周。

对照组每日给予安慰剂 2 粒，一日 3 次，温水冲服，连续服用 24 周。

4.药物发放

每个单位有连续编号的药物，受试者筛选期结束后，研究者按其入选先后顺序以药物编号递增方式发放，不得跳跃号发放。研究者于受试者就诊时开具处方，处方上写明药物编号。试验用药品管理员应按试验流程图发放试验药物，同时及时、准确在《临床试验药物使用登记表》上记录相关信息。导入期、筛选期结束后、第 12 周发放药物。

5.药物回收

药品管理员必须回收药物的外包装(包括包装盒、空袋)及剩余的研究药物，

同时在原始记录和 CRF 中记录受试者接受、使用和归还的药品数量。一旦发现有剩余药物或外包装没有归还，研究者都必须详细记录原因，以防类似情况再次发生。

6.合并用药的规定

6.1 禁用药物

用于治疗冠心病的中药及中成药，或对治疗冠心病疗效有影响的中药及中成药。

6.2 西医用药

两组患者均按照指南进行现代西医常规治疗，包括抗缺血（β受体阻滞剂、钙拮抗剂）、抗血小板(阿司匹林合/或氯吡格雷)、抗凝(肝素或低分子肝素)及调节脂质代谢（他汀类）药物，对于合并症如糖尿病、高血压、血脂异常、心衰、低血压休克等，应根据相关指南给予对症处理。合并用药或一直使用的针对其他疾病的给药方案应保持不变。研究者必须详细记录注明药品名称，剂量、用法、用药原因及用药时间。

6.3 合并用药的随访记录

所有的合并用药都必须详细记录在原始记录（门诊病历/住院病历）和 CRF 中，包括治疗药物名称、每日总剂量、使用原因、用药开始日期、停药日期。

7. 试验指标观测流程图

研究阶段		导入期（7天）	治疗期（用药期）			随访期（用药后）	
			治疗开始	第 12 周	第 24 周	第 12 周	第 24 周
签署知情同意书		√					
入选/排除标准		√					
问诊	一般情况	√					
	现病史	√					
	既往病史	√					
	合并用药情况	√					
查体	舌、脉	√	√	√	√	√	√
	生命体征	√	√	√	√	√	√
	体格检查	√	√	√	√	√	√
安全性观察	静息心电图		√	√	√	√	√
	血尿便常规		√	√	√	√	√
	肝功、肾功		√	√	√	√	√
	凝血功能*		√	√	√	√	√
	不良事件		√	√	√	√	√
药物发放		√	√	√			
疗效观察	主要终点指标		√	√	√	√	√
	次要终点指标		√	√	√	√	√
	心功能指标**		√	√	√	√	√
	运动耐量***		√	√	√		
	炎症指标		√	√	√	√	√
	血脂指标****		√	√	√	√	√
	自评量表		√	√	√	√	√
	中医症状评分		√	√	√	√	√

注：*凝血功能包括凝血酶原时间、活化部分凝血活酶时间、纤维蛋白原和凝血酶时间；**心功能指标包括心脏彩超（左室舒张末期容积、右室舒张末期容积、射血分数）和 BNP； ***运动耐量指标主要包括平板运动耐量和诱发缺血时间（仅前 100 例患者），分别于服药开始、服药 12 周、服药 24 周进行；****血脂检查包括血浆总胆固醇、甘油三酯、低密度脂蛋白胆固醇、高密度脂蛋白胆固醇。

8. 终点指标

包括主要终点指标（全因死亡、心源性死亡、非致死性心肌梗死、血运重建术）和次要终点指标（因心功能不全或复发心绞痛入院、脑卒中、TIA 及其它血栓并发症）。研究全程观察终点事件发生情况。

9. 安全性指标

包括血、尿、便常规，肝功能（ALT、AST）、肾功能（Cr、BUN），凝血四项（凝血酶原时间、活化部分凝血活酶时间、纤维蛋白原和凝血酶时间）。

10. 运动耐量指标

运动耐量指标主要包括平板运动耐量和诱发缺血时间（仅前 100 例患者），分别于服药开始、服药 12 周、服药 24 周进行。每个中心前 5 例随机患者将进行平板运动试验以获得上述指标。

11.依从性观察

为保证受试者的依从性，应使受试者充分理解试验的意义及按时用药的重要性，研究人员需要求受试者详细记录用药日记。对未完全按照要求用药者要及时进行说服，并详细记录原因。

参加临床试验的人员应相对固定，必须认真学习和讨论临床试验方案及临床试验手册，统一记录方式与判断标准，并积极采取措施（通知复诊、进行随访），控制病例脱落

12.不良事件与不良反应

12.1 定义

不良事件：病人或临床试验受试者接受一种药品后出现的不良医学事件，但并不一定与治疗有因果关系。

严重不良事件：临床试验过程中发生需住院治疗、延长住院时间、伤残、影响工作能力、危及生命或死亡、导致先天畸形等事件。

12.2 不良事件的记录

研究者发现不良事件后，应立即记载在原始记录及病例报告表中，如不良事件属于严重不良事件、新的药品不良反应或者药品严重不良反应，需按照相应流程进行报告。

12.3 不良事件的判断

严重程度判断

轻度：受试者可忍受，不影响治疗、不需要特别处理，对受试者康复无影响。

中度：受试者难以忍受，需要撤药或做特殊处理，对受试者康复有直接影响。

重度：危及受试者生命，致死或致残，需立即撤药或作紧急处理。

Ⅳ期临床试验 AE 的关联性判断参照 6 级判定标准

	1	2	3	4	5
肯定	＋	＋	＋	＋	－
很可能	＋	＋	＋	?	－
可能	＋	±	±?	?	±?
可能无关	－	－	±?	?	±?
待评价	需要补充材料才能评价				
无法评价	评价的必须资料无法获得				

注：+表示肯定； -表示否定； ±表示难以肯定或否定； ？表示不明

12.4 不良事件转归

死亡：导致生命结束（须收集死亡原因和死亡时间）。

未痊愈/未缓解：AE 经治疗后，症状未改善/未恢复。

痊愈：AE 经治疗后，症状完全消失，且没有后遗症状。

症状消失但有后遗症：AE 经治疗后，症状已消失但留有后遗症（记录时应注明后遗症的名称或表现）。

缓解：AE 经治疗后，症状改善。

不详：情况不明，未进行跟踪观察、记录，或受试者拒绝

12.5 不良事件的报告

一般不良反应只需在原始病历及病例报告表中记录，无需特别报告。

严重不良事件应该在获知该事件的 24 小时内，填写严重不良事件报告表并向国家药监局、研究中心所在省/市药品监督管理局、研究中心的伦理委员会及申办者报告，并保留相应的书面记录。

13.数据录入

采用网上数据传输，将病人的全部病历信息录入电子病历信息平台，采用第三方数据盲法管理，专人负责数据，经盲态审核后台数据，通过第三方进行数据数理统计和数据挖掘分析。

各病例入选后，对于定期的随访数据，由协作单位指派专门数据录入员录入，数据录入员及时、准确地将研究病历中的数据录入 eCRF。为保证数据录入的准确性，本研究采用双录入。

（3）数据监查：监查员在各中心研究现场登陆数据管理系统的数据监查端，100%的核对电子 CRF 数据与研究病历等源数据的一致性，发现问题可随时在线

发出疑问，数据录入员下载疑问，研究者给予解答，数据录入员直接在疑问管理界面更新数据。必要时监查员可以重复发出疑问。

（4）数据库的锁定：所有受试者完成试验，病历全部录入系统，并经监查员审阅、数据管理员的审核无误后，由数据管理员对数据进行锁定。锁定后的数据文件原则上不可再做改动。如数据库锁定之后发现的问题，经确认后可在统计分析过程中进行修正，并作记录和说明。

（5）数据导出：数据全部锁定后，由数据管理员将其导入到指定数据库，交统计人员进行统计分析。
